# Residual proton line width under refocused frequency-switched Lee-Goldburg decoupling in MAS NMR†

**DOI:** 10.1039/d3cp00414g

**Published:** 2023-03-17

**Authors:** Kathrin Aebischer, Matthias Ernst

**Affiliations:** a Physical Chemistry, ETH Zürich, Vladimir-Prelog-Weg 2 8093 Zürich Switzerland maer@ethz.ch +41 44 632 43 66

## Abstract

Despite many decades of research, homonuclear decoupling in solid-state NMR under magic-angle spinning (MAS) has yet to reach a point where the achievable proton line widths become comparable to the resolution obtained in solution-state NMR. This makes the precise determination of isotropic chemical shifts difficult and thus presents a limiting factor in the application of proton solid-state NMR to biomolecules and small molecules. In this publication we analyze the sources of the residual line width in refocused homonuclear-decoupled spectra in detail by comparing numerical simulations and experimental data. Using a hybrid analytical/numerical approach based on Floquet theory, we find that third-order effective Hamiltonian terms are required to realistically characterize the line shape and line width under frequency-switched Lee-Goldburg (FSLG) decoupling under MAS. Increasing the radio-frequency field amplitude enhances the influence of experimental rf imperfections such as pulse transients and the MAS-modulated radial rf-field inhomogeneity. While second- and third-order terms are, as expected, reduced in size at higher rf-field amplitudes, the line width becomes dominated by first-order terms which severely limits the achievable line width. We expect, therefore, that significant improvements in the line width of FSLG-decoupled spectra can only be achieved by reducing the influence of MAS-modulated rf-field inhomogeneity and pulse transients.

## Abstract 2

We analyze the source of the residual line width in refocused homonuclear-decoupled spectra in detail by comparing numerical simulations and experimental data. We find that rf imperfections can severely limit the achievable decoupling performance.

## Graphical abstract

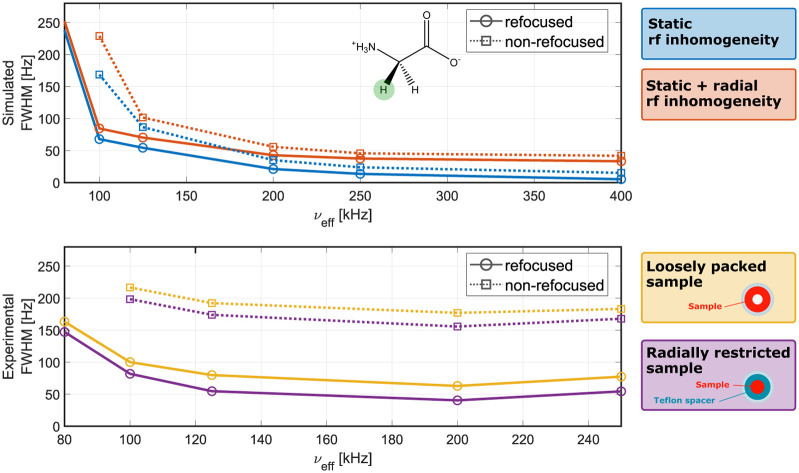

## 1. Introduction

Proton line widths in solid-state NMR experiments under fast magic-angle spinning (MAS) or homonuclear decoupling are still significantly larger than typical solution-state proton lines. The observed broadening in the spectra can predominantly be attributed to strong proton–proton dipolar couplings that are not completely averaged by experimental techniques available today and a distribution of chemical shifts due to the rf-field inhomogeneity in homonuclear decoupling. This lack of spectral resolution masks the wealth of information contained in proton chemical shifts and limits the application of proton-detected solid-state NMR.^1,2^ Further reduction in the line width by imporoved homonuclear decoupling schemes would allow a broader use of proton solid-state NMR in applications especially for small molecules and pharmaceuticals.^3–5^

The total observed line width in experimental NMR spectra is determined by several different contributions. Following the convention introduced by Maricq and Waugh,^6^ one usually distinguishes the inhomogeneous and the homogeneous broadening. The inhomogeneous contribution arises due to field and sample heterogeneities, such as shim imperfections, susceptibility differences over the sample, or a distribution of isotropic chemical shifts, and can be refocused completely by a Hahn echo.^7^ The homogeneous contribution on the other hand is only partially refocused by an echo and is comprised of a coherent and an incoherent part. Incoherent broadening arises from stochastic modulations of the local magnetic field due to molecular motion and chemical exchange,^8^ whereas the coherent contribution originates from spin–spin and spin–field interactions that are described by the spin-system Hamiltonian.

In homonuclear-decoupled spectra, a large part of the inhomogeneous contribution can be attributed to a distribution of effective isotropic chemical shifts arising from the static rf-field inhomogeneity.^9^ Variations of the rf-field amplitude over the sample volume lead to different tilt angles of the effective field which in turn impacts the scaling of the isotropic chemical shift. Such broadenings are characterized by an asymmetric shape of the line in the direction pointing away from the carrier position. These effects are typically large and dominate the observed shape and width of the spectral lines. A physical restriction of the sample to the center of the rotor improves the line shape significantly since the parts of the sample that show the strongest deviation from the optimum rf-field amplitude are eliminated.^9^ Selective pulses in the rotating frame^10,11^ represent an even better way to improve the line shapes by restricting the distribution of the rf-field. Such an approach allows the selective manipulation of the part of the sample that experiences a narrow band of rf-field values.

**Fig. 1:**
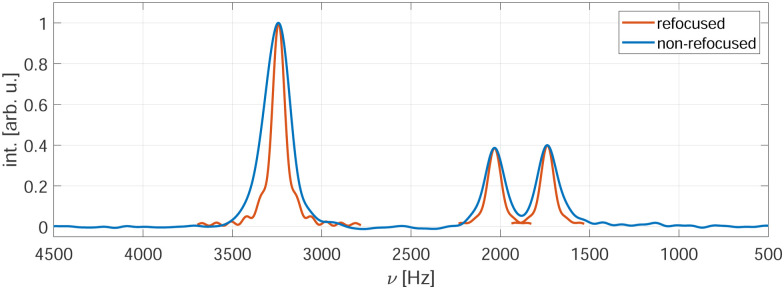
Experimental spectrum (blue) of glycine under FSLG decoupling using indirect detection. For comparison the refocused lines (red) are plotted at the spectral positions of the corresponding peaks. The two spectra were recorded with the pulse sequences shown in Fig. 2 using an effective rf-field amplitude of 125 kHz and a spinning frequency of 14 kHz. A 350 µs *e*-SNOB pulse in the spinlock frame was used to restrict the sample to areas experiencing rf field amplitudes close to the nominal value.

Even in such restricted samples, a spin-echo sequence leads to an additional significant reduction in the line width by refocusing inhomogeneous contributions.^12^ A typical experimental example of a spectral line width in an indirectly detected spectrum compared to the line shape under spin-echo detection is shown in Fig. 1 for a sample of glycine under frequency-switched Lee-Goldburg (FSLG) decoupling with an effective field strength of 125 kHz and a MAS frequency of 14 kHz. No rescaling of the chemical-shift axis was performed. Despite using a 350 µs *e*-SNOB pulse in the rotating frame to restrict the sample and thus reduce the rf-field inhomogeneity, the spin-echo decays (red) are significantly narrower than the lines in the spectrum (blue). This implies that there is still a considerable inhomogeneous contribution to the line width in the non-refocused spectra.

The aim of this paper is to better characterize the residual line broadening in echo-refocused homonuclear-decoupled spectra as well as the inhomogeneous contribution to the line width in strongly restricted samples. Potential sources for the observed inhomogeneous line broadening are the remaining rf-field inhomogeneity, sample properties like the susceptibility broadening or imperfect shim and magic-angle adjustments. While some of these contributions (rf-field inhomogeneity, pulse transients) can be included in numerical simulations, the magnitude of other contributions can only be inferred from comparing simulations with experimental measurements. Using numerical simulations, we are able to reproduce the experimental refocused line width which allows us to roughly estimate the magnitude of the various contributions to the residual line width. The coherent contribution to the line width is characterized using an analytical treatment based on Floquet theory.^13–15^ We chose to investigate the simple non-super-cycled FSLG decoupling sequence as it can easily be described analytically and allows the separation of different orders even when experimental imperfections are included.

## 2. Theory

In proton-detected solid-state NMR experiments, MAS alone is usually not sufficient to average out the strong proton–proton dipolar couplings unless spinning frequencies exceeding 100 kHz are used.^2,16^ To achieve such fast MAS, rotors with outer diameters around 0.7 mm and below are required, leading to small sample amounts and corresponding low sensitivity. Therefore, homonuclear-decoupling techniques are often used in combination with MAS at intermediate to slow spinning frequencies. One commonly used family of decoupling sequences is based on the work of Lee and Goldburg.^17,18^ In the original Lee-Goldburg (LG) scheme, off-resonance rf irradiation is used to generate an effective field *ω*^LG^_eff_ that is tilted with respect to the *B*_0_-field. If the resonance offset Δ*ω* and the rf-field amplitude *ω*_1_ are adjusted such that the tilt angle1
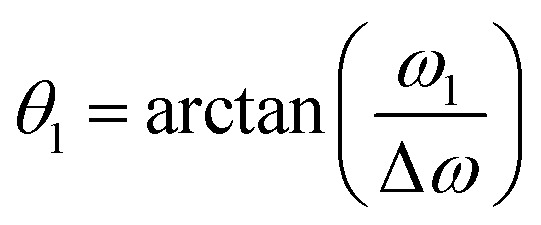
is equal to the magic angle *θ*_m_ ≈ 54.736°, the homonuclear dipolar couplings are averaged out in a first-order approximation while the isotropic chemical shift is scaled by a factor of cos(*θ*_m_) ≈ 0.577. In frequency-switched Lee-Goldburg (FSLG) decoupling,^19^ better compensation of higher-order terms is obtained by switching the sense of rotation after every full 2π-rotation about the effective field. This is achieved by a phase inversion of the rf and a simultaneous sign change of the offset. The FSLG scheme is usually implemented using phase-modulated on-resonance irradiation with a phase-modulation frequency of |Δ*ω*|. The frequency switching can then be realized by inverting the sense of rotation of the phase. An example of such a phase ramp for an effective field strength of 125 kHz along the magic angle is shown in Fig. S8 in the ESI.† Other pulse sequences based on the LG scheme include phase-modulated LG (PMLG*n*) sequences,^20^ where a coarsely discretized phase ramp with *n* steps is used instead of a continuous modulation and the DUMBO family of sequences^21^ that was developed with the help of numerical optimization.

### 2.1 Floquet description

The coherent contribution to the line width can be characterized by the spin-system Hamiltonian that contains all relevant spin–spin and spin–field interactions. In the high-field approximation, the Hamiltonian for a homonuclear spin system consisting of *N* protons subject to rf irradiation under MAS is given by2
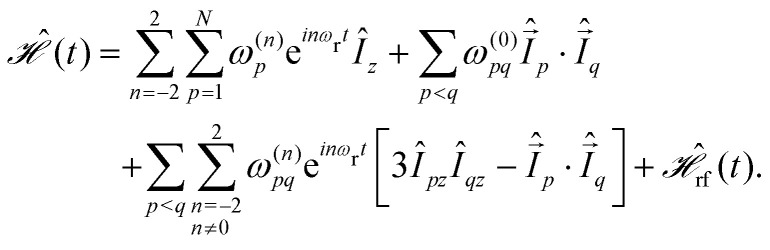
The Fourier components *ω*^(*n*)^ of the spatial tensors are given by

3 *ω*^(0)^_*p*_ = *Ω*_*p*_

4
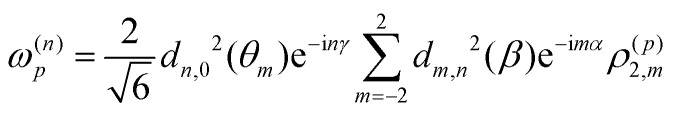

5 *ω*^(0)^_*pq*_ = 2π*J*_*pq*_

6
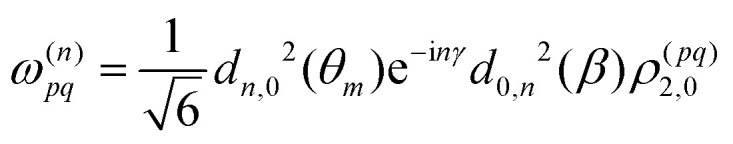
for the isotropic and anisotropic chemical shifts of a spin *I*_*p*_, the scalar *J* and the anisotropic dipolar coupling between two spins *I*_*p*_ and *I*_*q*_. The orientation of the tensors in the rotor-fixed frame is described by the Euler angles (*α*,*β*,*γ*). Tensor elements in the principal axis system are denoted as *ρ*_*l*,*m*_ and *d*^*ℓ*^_*m,m*'_(*β*) correspond to the reduced Wigner matrix elements.

For FSLG decoupling, all spins are irradiated with a constant rf-field amplitude modulated by a linear phase ramp. The rf Hamiltonian can, therefore, be written as7
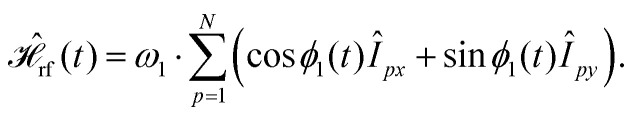
Deviations of the rf-field amplitude *ω*_1_ and phase *ϕ*_1_(*t*) from the nominal values due to experimental imperfections such as the rf-field inhomogeneity and pulse transients can be included in 
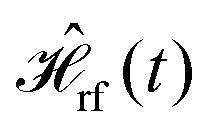
 using time-dependent coefficients as8
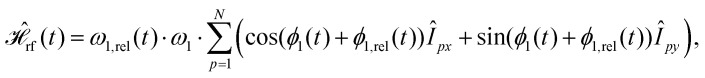
where additional time-dependent parameters for the relative rf-field amplitude *ω*_1,rel_(*t*) and phase *ϕ*_1,rel_(*t*) have been introduced. They can originate from the MAS modulation of the phase and amplitude due to the radial contribution to the rf inhomogeneity.^22^ In this case, *ω*_1,rel_(*t*) and *ϕ*_1,rel_(*t*) will be periodic with the rotor frequency *ω*_r_ and the rf Hamiltonian will only have a period of finite length if the modulation frequency of the pulse sequence and the MAS frequency are commensurate. Including the radial rf inhomogeneity in a theoretical treatment therefore requires *cω*_m_ = *ω*_r_, where *c* is an integer that should be greater than four in order to avoid resonance conditions up to and including second order.

The total-time-dependent Hamiltonian in eqn (2) can be transformed into an interaction frame with respect to 
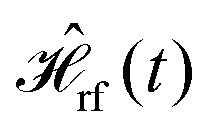
9
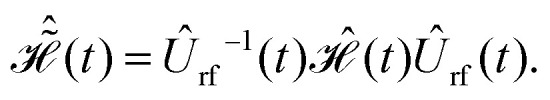
The propagator characterizing this interaction-frame transformation is given by10
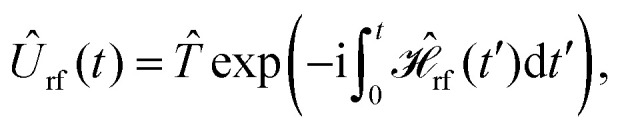
where the proper time ordering of non-commuting terms is ensured by the Dyson time-ordering operator *T̂*.^23^ For a rf Hamiltonian that is periodic with *ω*_m_ the *Î*_*z*_ spin operators in eqn (2) transform according to

11 *Ĩ̂*_*z*_(*t*) = *Û*_rf_^−1^(*t*)*Î*_*z*_*Û*_rf_(*t*)

12
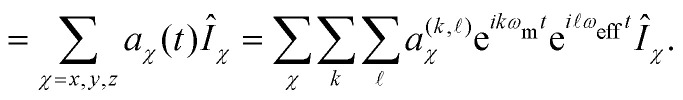
This interaction-frame trajectory is characterized by two basic frequencies: the modulation frequency of the pulse scheme *ω*_m_ and an additional effective field *ω*_eff_ that arises if the propagator over a full rf cycle is not unity. The additional field can be computed from the overall flip angle over one rf period *ω*_eff_ = *β*_eff_/*τ*_m_.^24^ The Fourier coefficients *a*^(*k*,*ℓ*)^_*χ*_ are independent of the details of the spin system and fully describe the interaction-frame trajectory.^14,25^ An ideal FSLG rf cycle corresponds to two 2π-rotations in opposite directions about the effective field *ω*^FSLG^_eff_. Thus, the modulation frequency is given by *ω*^FSLG^_eff_/2 and the additional effective field is zero. However, experimental imperfections of the rf irradiation can lead to deviations from the ideal trajectory and thus non-zero values of *ω*_eff_. Since the effective fields are often small compared to the other two frequencies, the 
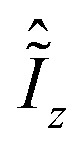
 spin operators can further be transformed into a second interaction frame with 
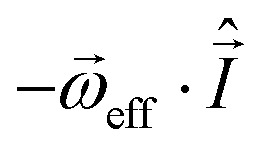
.^26^ The interaction-frame trajectory can now be characterized by a single frequency13
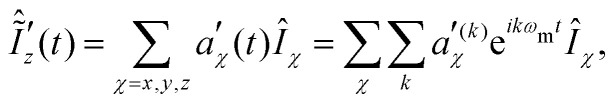
with the new Fourier coefficients 
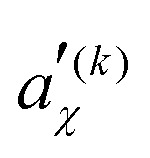
. The resulting Hamiltonian can then be written in terms of only two basic frequencies14
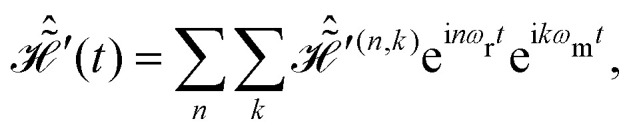
with Fourier components15
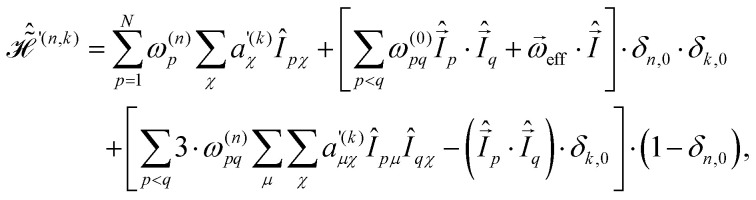
where the two-spin Fourier coefficients 
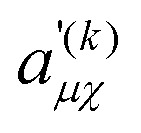
 were introduced for a more convenient notation. They can be computed as the convolution of single-spin coefficients16
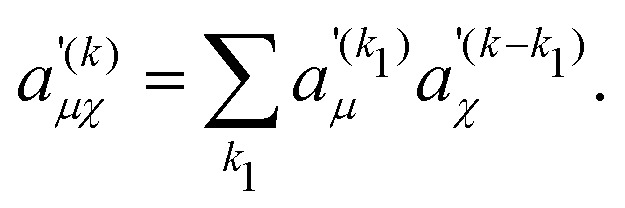
Expressions for the time-independent effective Hamiltonian can now be derived using bimodal Floquet theory. To first order, all terms satisfying the resonance condition

17 *n*_0_*ω*_r_ + *k*_0_*ω*_m_ = 0

will contribute. The first-order effective Hamiltonian is then given by the sum of resonant and non-resonant (*n*_0_ = *k*_0_ = 0) Fourier components18
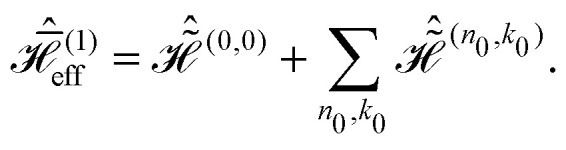
In full analogy, the second-order effective Hamiltonian is given by19
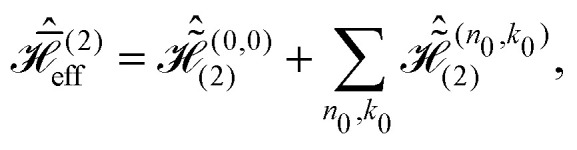
where20
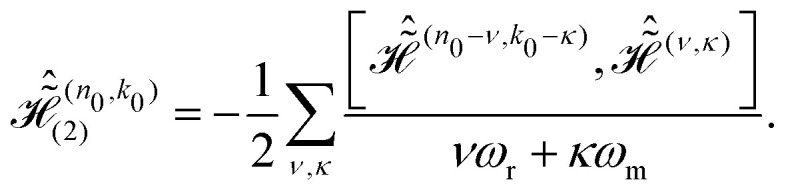
Likewise, the third order effective Hamiltonian can be written as21
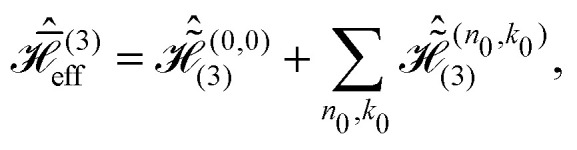
with22
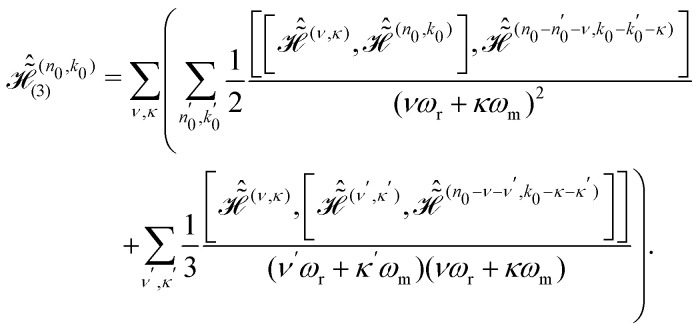
Summations over indices *ν*, *κ* and *ν*′, *κ*′ in eqn (20) and (22) are restricted to values satisfying *νω*_r_ + *κω*_m_ ≠ 0 and *ν*′*ω*_r_ + *κ*′*ω*_m_ ≠ 0.

## 3. Methods and materials

### 3.1 Numerical simulations

The contributions to the residual coherent line width under homonuclear FSLG decoupling were studied using numerical simulations of spin systems with six to eight protons with parameters based on the crystal structure of glycine (α-polymorph). Details of the spin-system parameters are given in Tables S7 and S8 in the ESI.† Numerical simulations were performed using the GAMMA spin-simulation environment^27^ at a *B*_0_-field of 14.1 T. Simultaneous averaging over all three powder angles was implemented according to the ZCW scheme^28^ using 1154 crystallite orientations. In order to reduce the computational cost, a MAS frequency of 12.5 kHz was used leading to the synchronization of the FSLG decoupling and the MAS rotation after five FSLG cycles and thus allowing a reuse of propagators. The propagator *Û*_tot_ over a rotor period was computed by time-slicing the full time-dependent homonuclear Hamiltonian (see eqn (2)) with a time-resolution of 50 ns. For Hahn echo simulations, the total echo propagator was then computed as *Û*_echo_ = *Û*^*m*^_tot_*Û*_π,*y*_*Û*^*m*^_tot_, where *Û*_π,*y*_ corresponds to an ideal *δ*–π-pulse and *m* is an integer representing the incrementation of the echo time *τ* = *m*·*τ*_r_. The initial density operator was set to *F̂*_*y*_ and the phase-sensitive detection operator tilted by the magic angle as *Î*_det_ = *Î*_*y*_ + i·(sin(*θ*_m_)*Î*_*z*_ + cos(*θ*_m_)*Î*_*x*_). For the Hahn-echo decay curves, a single-channel detection using *Î*_det_ = *Î*_*y*_ was used. All spins were detected individually.

The rf-field inhomogeneity was included in the rf Hamiltonian by introducing the relative rf-field amplitude *ω*_1,rel_(*r⃑*,*t*) and phase *ϕ*_1,rel_(*r⃑*,*t*) as defined in eqn (8) that depend on the position of the crystallite in the sample space *r⃑* and the rotor orientation. Their time-dependence is periodic with the MAS frequency and arises due to the radial contribution to the rf-field inhomogeneity.^29,30^ The rf field distribution in a 1.9 mm rotor (see Fig. S1 in the ESI†) was computed based on physical models of the solenoid coil^31,32^ and used as input for numerical simulations. In order to separate the effects of the rf-field modulations due to the radial rf-field inhomogeneity from the static contribution, two distinct cases were studied^22^

• C1: static and radial rf-field inhomogeneity: time-dependent amplitude, time-dependent phase *ω*_1,rel_(*r⃑*,*t*), *ϕ*_1,rel_(*r⃑*,*t*)

• C2: static rf-field inhomogeneity only: time-averaged constant amplitude, zero phase 
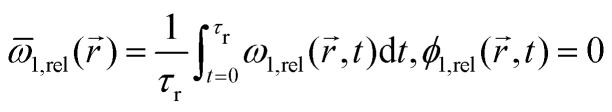


Individual volume elements in the 1.9 mm rotor were simulated separately and simulation results summed during data processing. The spatial resolution was set to 0.05 mm for *r* and *z* (total of 1936 volume elements) and the *rz*-plane starting at *ϑ*(*t* = 0) = 0° was simulated. In order to account for the increase in sample volume with radial distance and the coil sensitivity (reciprocity theorem^32,33^), volume elements were weighted with *r* and the average of the relative rf-field amplitude over one rotor cycle *ω̄*_1,rel_. A more detailed description of the treatment of the rf-field inhomogeneity including the time-dependent modulations of the relative rf-field amplitude and phase can be found in ref. 22.

Simulations of effective Hamiltonians up to order *n* were performed by implementing the commutator expressions for 
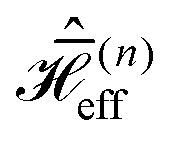
 given in Section 2.1 (eqn (20) and (22)). The propagators for these time-independent effective Hamiltonians can then easily be calculated as 
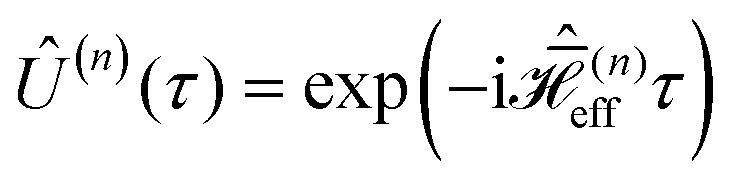
. Fourier coefficients characterizing the interaction-frame trajectory of single- and two-spin operators, 
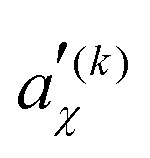
 and 
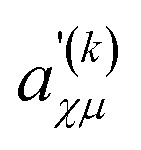
 respectively (see Section 2.1, eqn (13)), were computed in Matlab (The MathWorks Inc., Natick, MA, USA.) and given as input to the simulations along with the strength and orientation of the additional effective field 
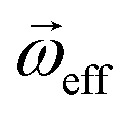
.

Data processing was done in Matlab using home-written routines. The simulated FIDs of the single-spin detection were zero filled to twice the number of data points and a cosine-squared apodization applied prior to Fourier transform. Line widths were determined as FWHM from the simulated spectra. Simulated dephasing curves (single-spin detection) were normalized to the first datapoint and an exponential decay 
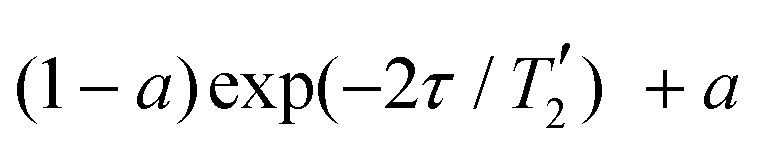
 fitted to the real part in order to extract the refocused FWHM as 
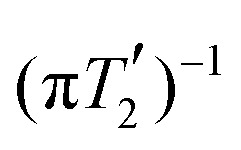
.

### 3.2 Experimental

Experimental non-refocused and refocused proton line widths of natural abundance glycine were measured on a 500 MHz Bruker Avance III HD NMR spectrometer equipped with a Bruker 1.9 mm triple-resonance MAS probe in double-resonance configuration at a MAS frequency of 14 kHz. All measurements were performed at a set temperature of 285 K. Powdered glycine was packed in three different ways into rotors: (i) a fully packed 1.9 mm rotor, (ii) a loosely packed rotor containing *ca.* 20% less sample and (iii) a rotor containing a cylindrical Teflon spacer that allows the spatial restriction of the sample space in the radial direction. The loosely packed rotor was spun up manually to 4 kHz and the spinning frequency slowly increased to 30 kHz without any instabilities. It was then spun at 30 kHz for 48 h in order to ensure that the sample was compacted and stably packed close to the rotor wall. The resulting sample distribution was observed under a microscope, revealing that a hole without sample (diameter roughly 0.6 mm) resulted in the center of the rotor. For the third rotor, a tube-shaped Teflon spacer with a central hole diameter of 0.8 mm was inserted into the 1.9 mm rotor. The sample is therefore restricted to a cylindrical space in the center of the rotor with a diameter of 0.8 mm. A schematic depiction of the sample distribution in the loosely packed and the radially restricted rotor is shown in Fig. S3 in the ESI.†

**Fig. 2:**
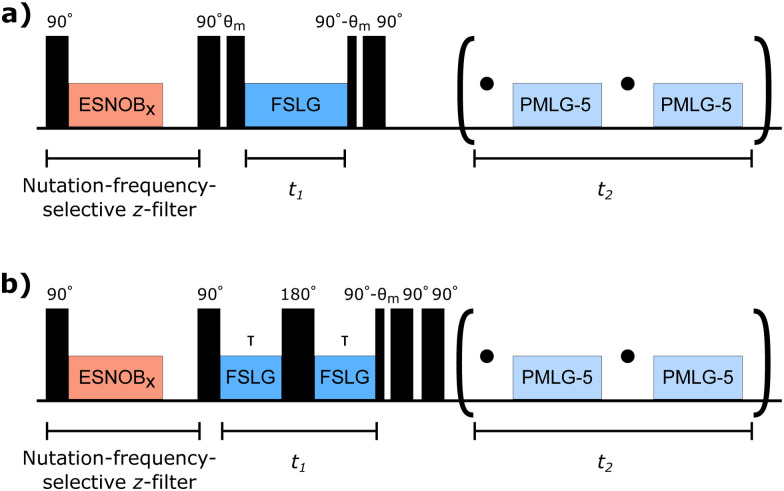
Schematics of the pulse sequences used in this work. (a) Non-refocused FWHM were measured as 2D proton–proton chemical shift correlation experiments with FSLG decoupling in the indirect dimension. (b) Refocused FWHM were measured in pseudo-2D experiments with a Hahn echo under FSLG decoupling in *t*_1_. For both pulse schemes, chemical shift resolution in the direct dimension is achieved using wPMLG detection with a pure *z*-rotation. The nutation-frequency-selective *z*-filter at the beginning allows the selective excitation of part of the rf-field distribution.

Non-refocused line widths were measured in 2D proton–proton chemical-shift correlation spectra with FSLG decoupling in the indirect dimension. For the refocused line widths, 2D spectra with a Hahn echo under FSLG decoupling during *t*_1_ were recorded. In both cases, chemical-shift resolution in the direct dimension was achieved using windowed PMLG (wPMLG) detection with a pure *z*-rotation.^34^ Schematics of the pulse sequences are shown in Fig. 2 (pulse programs can be found in the ESI†). In the direct dimension, PMLG-5 decoupling with an effective field of 125 kHz along the magic angle was used and 1536 complex data points were acquired with a spectral width of 49 020 Hz. In the indirect dimension FSLG decoupling with effective-field strengths between 80 and 250 kHz was applied using shaped pulses with a time resolution of 100 ns. A time increment of 48 µs was used in *t*_1_ and 128 points recorded with eight scans each. States-type data acquisition^35^ was used for phase-sensitive detection and sign discrimination in *t*_1_ for measurements of non-refocused line widths. The carrier position during *t*_2_ was experimentally optimized in 1D wPMLG-5 spectra. During *t*_1_, the carrier was positioned outside the spectral region of interest. All power levels were calibrated using nutation spectra.

Based on our previous work on nutation-frequency-selective inversion pulses,^11^ we implemented a nutation-frequency-selective *z*-filter, enabling the restriction of the sample in terms of the rf field amplitude experienced. Such a *z*-filter consists of a hard 90° excitation pulse followed by a selective 90° or 270° pulse in the spinlock frame that rotates the magnetization of the desired part of the rf-field distribution back onto the *z*-axis. During the dephasing delay, the remaining transverse magnetization decays and all following pulses will only affect the selected part of the sample. This nutation-frequency-selective *z*-filter can be used in combination with any pulse scheme to allow the selective excitation of a part of the rf-field distribution. Any band-selective excitation pulse can be used in principle, but we chose the *e*-SNOB family^36^ of selective pulses in this work. A spin-lock amplitude of 100 kHz and a 350 µs *e*-SNOB pulse (corresponding to a 4 kHz bandwidth) in the spinlock frame with a modulation frequency of 100 kHz and a dephasing delay of 2 ms were used. A simulation of the excitation profile for such a pulse as well as example nutation spectra with and without the selective excitation can be found in Fig. S2 in the ESI.†

**Fig. 3:**
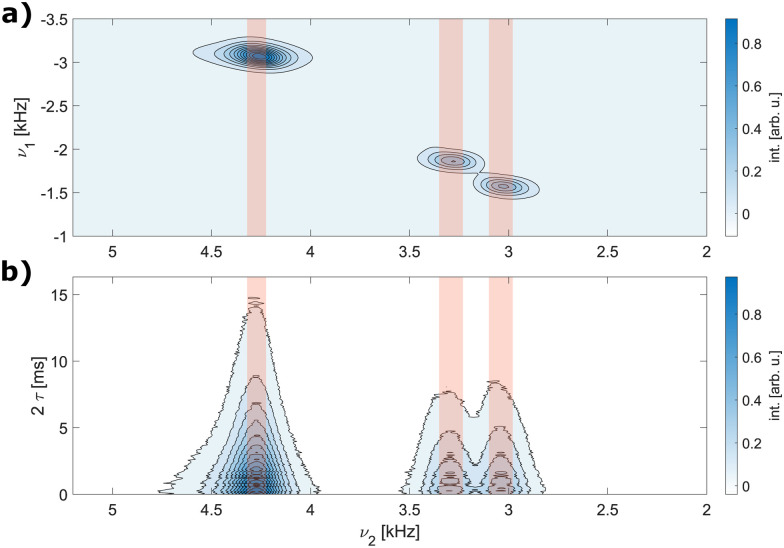
Experimental non-refocused 2D proton spectrum (a) and pseudo-2D dataset (b) of natural abundance glycine (fully packed rotor) measured at 500 MHz proton Larmor frequency using a 1.9 mm probe and a MAS frequency of 14 kHz. The corresponding pulse sequences are shown in Fig. 2. No chemical-shift scaling was applied. The integration ranges in the direct dimension are set to half of the FWHM of the first *t*_1_ increment and are indicated in red. Further details on the determination of the non-refocused and refocused FWHM from such datasets can be found in Fig. S4 and S5 in the ESI.†

Data processing was done in Matlab using home-written routines. In the direct dimension (and the indirect dimension for measurements of non-refocused line widths), zero-filling to 4096 points was applied. Non-refocused FWHM were determined from 1D spectra obtained from separate summation over each of the peaks in the direct dimension. The width of the summation range was set to half of the FWHM of the spectral line in the direct dimension (see Fig. 3a). The FWHM were determined directly and no chemical-shift scaling was applied. For the refocused line widths, the integrated intensity of each resonance (same integration range, see Fig. 3b) was determined as a function of the echo time *τ* and an exponential decay 
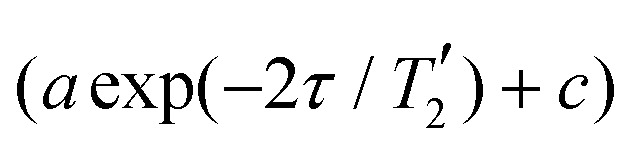
 fitted and the refocused FWHM in Hz computed as 
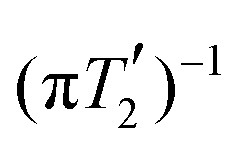
. Examples of experimental 2D spectra and pseudo-2D datasets are shown in Fig. 3. Further details concerning the data processing can be found in Fig. S4 and S5 in the ESI.†

## 4. Results and discussion

### 4.1 Effects of RF-field Inhomogeneity on the line width

**Fig. 4:**
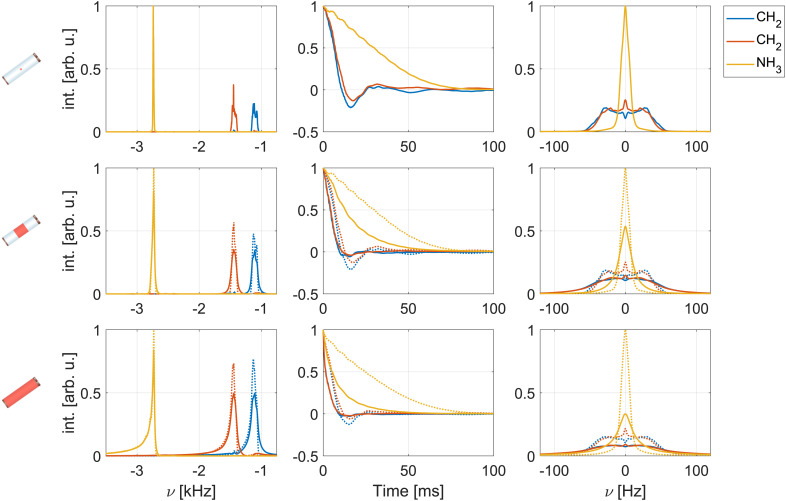
Simulated spectra (left) and dephasing curves (middle as well as the Fourier transform on the right) for the three central protons in a six-spin system based on glycine under FSLG decoupling. The effective field strength was set to 125 kHz and a MAS frequency of 12.5 kHz assumed. Shown are simulations for the central volume element (no rf-field inhomogeneity, top row) as well as the central third (middle row) and the full sample space of a 1.9 mm rotor (bottom row). Solid lines correspond to C1 (both static and radial rf-field inhomogeneity taken into account, see Section 3.1), dotted lines to C2 (only static rf-field inhomogeneity). Modulations of the rf-field amplitude and phase due to the radial rf-field inhomogeneity lead to line broadening and shortened dephasing times in echo simulations.

Examples of simulated spectra and Hahn-echo dephasing curves under FSLG decoupling are shown in Fig. 4. An effective field strength of 125 kHz and a MAS frequency of 12.5 kHz were used, avoiding all resonance conditions up to and including second order. Third-order resonance conditions are possible for these parameters but are typically small. The top row shows results for a perfectly homogeneous and ideal rf field, while the middle and bottom row show simulations for the central third and the full sample space of a 1.9 mm rotor including the rf-field inhomogeneity (see Fig. S1 in the ESI† for the spatial rf-field distributions). Solid lines correspond to simulations where both the static and the MAS-modulated radial rf-field inhomogeneity were taken into account (C1, see Section 3.1), dotted lines indicate simulations considering only the averaged static rf-field inhomogeneity (C2). Refocused and non-refocused line widths were extracted from numerical simulation as described in Section 3.1 and are summarized and compared with experimental results for a fully packed rotor in Table S1 in the ESI.†

As expected, simulated spectra (left column in Fig. 4) show significantly broader lines and a typical asymmetric foot when the full sample space is considered. This indicates that the line width is dominated by the static rf-field inhomogeneity that leads to a distribution of chemical shift scaling factors.^9^ An additional broadening is observed when the MAS-modulated rf-field inhomogeneity is taken into account (solid lines).^22^ Under a spin-echo sequence (middle and right column of Fig. 4), the distribution of chemical shifts due to the static rf inhomogeneity is refocused and similar line widths result for a perfectly homogeneous rf field and simulations including the static rf inhomogeneity in the central third or the complete 1.9 mm rotor (dotted lines). A substantially more rapid dephasing in addition to the damping of the oscillating component is observed when rf-field amplitude and phase modulations (C1) are taken into account for the central third or the full rotor. Comparison of the simulated FWHM (see Table S1 in the ESI†) for C1 (static and radial rf inhomogeneity) and C2 (static rf inhomogeneity only) shows that rf-field amplitude and phase modulations due to the radial rf inhomogeneity lead to 20–30 Hz broadening for the restricted sample space. In the full sample, the broadening is significantly stronger (up to 60 Hz for refocused line widths) as the strength of the modulations increases towards the edges of the sample space (see Fig. S1 in the ESI†). Simulations for an effective field of 250 kHz show that this broadening effect is even more pronounced at higher decoupling field strengths (see Fig. S7 and Table S2 in the ESI† for resulting spectra and FWHM at 250 kHz). These observations indicate that the decoupling performance is considerably deteriorated by the radial rf-field inhomogeneity especially at higher rf-field strengths.

**Fig. 5:**
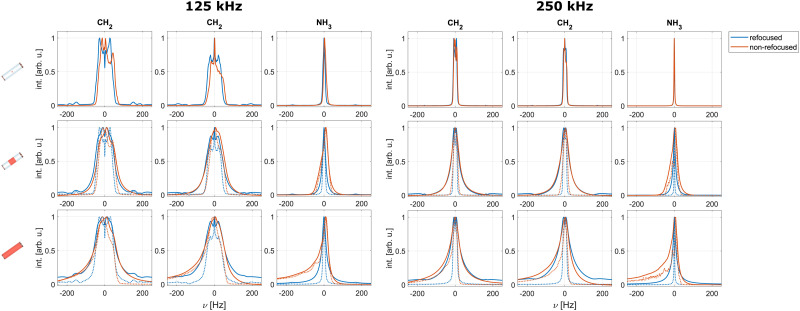
Comparison of simulated refocused and non-refocused line shapes under FSLG decoupling for the three central spins in a six-spin system based on glycine. Shown are simulations for effective field strengths of 125 kHz (left panel) and 250 kHz (right panel). A MAS frequency of 12.5 kHz was assumed in both cases. Simulations in the top row assume a perfectly homogeneous rf field, whereas results in the middle and bottom row take the rf-field inhomogeneity in the central third and the full sample space of a 1.9 mm rotor into account. Solid lines correspond to simulations taking the full rf inhomogeneity (radial and static, C1) into account, while only the static rf inhomogeneity (C2) was considered for dotted lines. All spectra were processed with 2 Hz additional exponential line broadening.

Fig. 5 shows a comparison of the simulated refocused and non-refocused line shapes for effective field strengths of 125 kHz and 250 kHz. Lines from non-refocused simulations were shifted by the scaled isotropic chemical shift (cos(*θ*_m_)·*Ω*_*p*_ ≈ 0.577·*Ω*_*p*_) and both simulations were normalized to their respective maximum intensity. Refocused lines are always symmetric about the origin while the non-refocused lines can show asymmetry. For both methylene protons, the obtained refocused and non-refocused line widths appear very similar, indicating that only a small part of the coherent contribution is refocused. At an effective field strength of 125 kHz we can again see a broadening of the lines by 20–30 Hz in the restricted sample if the MAS-modulated rf-field inhomogeneity is included in the simulations (solid *vs.* dotted lines in the center row of Fig. 5). Differences between the line shapes can mainly be observed in simulations of the full rotor where the asymmetric feet that arise due to the distribution of chemical-shift scaling factors^9,22^ are eliminated in the echo simulations. This effect is more pronounced for the amine proton that has a larger chemical shift and smaller dipolar couplings. Similar observations can be made for a higher field strength of 250 kHz (right panel in Fig. 5), but the broadening due to the MAS-modulated rf-field inhomogeneity is much more pronounced at higher rf-field amplitude. Compared to simulations at an effective field strength of 125 kHz there is a significantly larger difference between the line width observed for simulations with only the static rf inhomogeneity (C2, dotted lines) and those including the radial contribution to the rf inhomogeneity (C1, solid lines) at 250 kHz. In contrast to these observations, the obtained values for the FWHM (see Table S1 in the ESI†) show significant differences (approx. 30 Hz) between the refocused and the non-refocused line widths. While this would be expected due to the distribution of chemical-shift scaling factors^9,22^ for the simulations of the full rotor and to some extent for the simulations of the central third of the rotor, differences are also observed for simulations of the central volume element where the line widths (see Fig. 5, top row) are almost identical. This shows that characterizing the refocused line width by a single number is challenging, since the simulated dephasing curves are not necessarily represented well by an exponential decay especially in simulations with a limited number of spins. Extracting the refocused FWHM from the fitted 
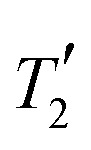
 is, therefore, not necessarily reliable and the numbers given in Table S1 in the ESI† can only give a rough estimate of the true line width.

**Fig. 6:**
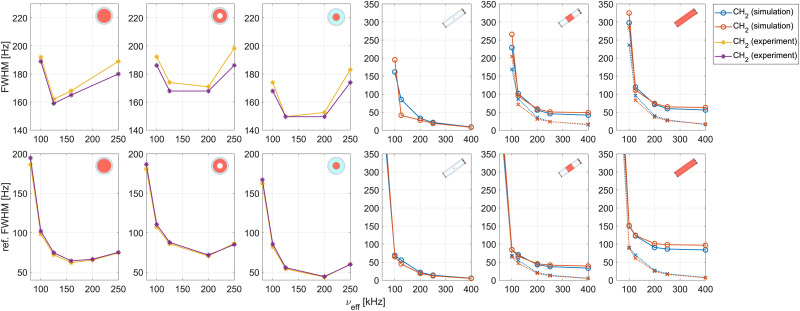
Dependence of experimental and simulated non-refocused (top row) and refocused (bottom row) FWHM on the rf-field strength during the FSLG decoupling for the two methylene protons in glycine. Experimental FWHM are shown for a fully packed, a loosely packed and a radially restricted sample (first three columns). A nutation-frequency-selective *z*-filter was used for sample restriction along the rotor axis. Simulation results assuming a perfectly homogeneous rf field as well as taking the rf-field inhomogeneity of the central third and the full sample in a 1.9 mm rotor into account are shown (final three columns). The relevant sample space is indicated in red in the rotor schematics in each plot. Dotted lines indicate simulations where only the static rf-field inhomogeneity was considered (C2), solid lines indicate simulations taking the full rf inhomogeneity into account (C1). The different *y*-scales for the experimental and simulation results should be noted. Modulations of the rf-field due to the radial rf-field inhomogeneity limit the decoupling performance at high rf field strengths.

Experimentally, the non-refocused lines are approximately 100 Hz broader than the refocused ones (see Fig. 6), indicating that the inhomogeneous contribution to the line width (*e.g.*, shim, chemical-shift distribution, static rf-field distribution) is on the order of 100 Hz. As the distribution of the chemical-shift scaling factors due to the static rf inhomogeneity is the only inhomogeneous contribution taken into account in the simulations, the obtained non-refocused FWHM are significantly narrower than the experimental ones. The discrepancy between experiment and simulation for the refocused FWHM on the other hand is much smaller. For the strongly coupled methylene protons, simulated refocused line widths for the central third of the 1.9 mm rotor agree well with the experimental FWHM obtained using a nutation-frequency-selective *z*-filter for sample restriction. In contrast, the simulated line widths for the NH_3_ protons are significantly narrower than in the experiment. This is to be expected, as the NH_3_ protons are approximated by a single spin in the simulations neglecting the relatively strong intra-NH_3_ couplings. The couplings between the NH_3_ and CH_2_ protons are computed based on the average position of the three amine protons. When the simulation results for individual volume elements are weighted with the excitation efficiency of the 350 µs *e*-SNOB pulse in the spinlock frame (see Fig. S2 in the ESI†) used in experiments, instead of approximating the excited sample by the central third, a broadening of *ca.* 2–3 Hz and 5 Hz is observed for the non-refocused and refocused FWHM respectively (see Table S3 in the ESI†) resulting in even better agreement with experimental values.

Furthermore, simulations with additional spins were performed to study the effect of a larger dipolar coupling network. In simulations of an eight spin system (two additional NH protons) for example, the observed FWHM for the CH_2_ group, both refocused and non-refocused, were approximately 10 Hz broader than those obtained for the six-spin system (see Table S4 in the ESI†). More significant broadening is observed for the NH proton, since it is strongly coupled to the two additional spins. As the dipolar couplings dominate the coherent contribution to the line width, the choice of spin system parameters is crucial and small deviations in the proton–proton distances assumed in the simulations will have a strong impact on the obtained FWHM.

Fig. 6 shows experimental and simulated spectral (top row) and refocused (bottom row, from fitted 
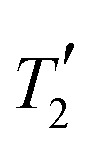
) FWHM for the CH_2_ protons in glycine as a function of the strength of the effective field during FSLG decoupling. The first three columns show experimental results for a full rotor, a loosely packed sample and a radially restricted sample. In all experiments, sample restriction along the rotor axis to reduce the effects of the static rf-field inhomogeneity is achieved using a nutation-frequency-selective *z*-filter. The corresponding experimental dephasing curves are shown in Fig. S6 in the ESI.† The final three columns show simulated FWHM for the central volume element (ideal and homogeneous rf field) as well as for simulations taking the rf inhomogeneity in the central third and the full sample space in a 1.9 mm rotor into account.

The experimental data clearly shows the detrimental effects of the radial rf inhomogeneity. The refocused and non-refocused line width obtained for the axially restricted sample are about 20 Hz narrower than those obtained for the loosely packed sample because the sample is located closer to the edge of the rotor where MAS modulations of the rf-field inhomogeneity are stronger. The line width obtained for the loosely packed sample are even approximately 10 Hz broader than those obtained for the fully packed rotor. Below an effective field strength of 100 kHz, strong broadening is observed for both simulation and experiment, as second-order resonance conditions are no longer avoided (*ν*_m_ = 50 kHz). This is in good agreement with expectations based on a theoretical treatment (see Section 2.1). In the experimental results for all three samples, strong broadening of the non-refocused FWHM is observed for decoupling field strengths exceeding 200 kHz. For the refocused line width, a decrease of the line width up to a rf-field amplitude of 200 kHz is observed with a slight increase of the FWHM for a 250 kHz effective field. This increase can potentially be attributed to other experimental rf imperfections such as pulse transients (see Section 4.2 below). In numerical simulations, the obtained refocused and non-refocused line widths decrease with increasing rf-field strength when an ideal rf-field or only the static rf-field inhomogeneity is considered (dotted lines, C2). However, when rf-field amplitude and phase modulations due to the radial rf inhomogeneity are taken into account (solid lines, C1), no further line narrowing is observed for rf-field strengths beyond 250 kHz. Such a plateau is reached in simulations of both the central third and the full sample space, but the difference between the two cases (C1 and C2) is larger for the full sample, as stronger modulations are encountered at the edges of the sample space. This indicates that the radial rf-field inhomogeneity is one of the reasons that limits the achievable refocused line width, especially at high decoupling field strengths. The simulation results agree well with the line broadening observed for the loosely packed rotor in the experimental data.

Comparing numerical simulations and experimental data we can conclude that the inhomogeneous broadening by shim imperfections (*B*_0_ inhomogeneity), susceptibility effects, or sample heterogeneity is on the order of 60–80 Hz for the strongly coupled methylene protons in our glycine sample. The inhomogeneous broadening due to the rf-field inhomogeneity in a sample restricted to the central third of the rotor is on the order of 10–30 Hz with a significantly larger contribution and a strong asymmetric foot in the full sample. It is important to remember that precise numbers are difficult to obtain since the line shapes are irregular especially in simulations of small spin systems and the obtained numbers depend on the details of how the line width is determined. For higher decoupling field strengths, the refocused line width appears to be limited by the radial rf field inhomogeneity.

### 4.2 Influence of pulse transients on the line width

In addition to the spatial rf-field inhomogeneity, deviations from ideal pulse shapes, commonly referred to as pulse transients,^37,38^ can affect the decoupling performance. These transient deviations during the rise and fall of an rf pulse inherently depend on the characteristics of the resonant circuit that is used and can be measured experimentally.^9,39,40^ For simple resonant circuits, analytical models have also been developed that can be used to characterize pulse transients. Following a simple model introduced in ref. 37, deviations from the ideal pulse *p*_ideal_(*t*) can be described by23
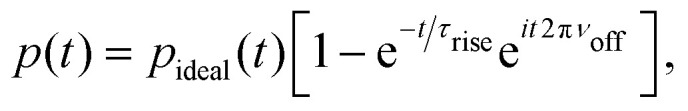
where the pulse transients are characterized by the pulse rise time *τ*_rise_ and the electronic frequency offset *ν*_off_. Pulse-transient effects on LG decoupling sequences have been discussed extensively in the literature^9,20,41^ and their influence on the refocused and non-refocused FWHM in combination with the rf-field inhomogeneity is studied here only in limited detail.

**Fig. 7:**
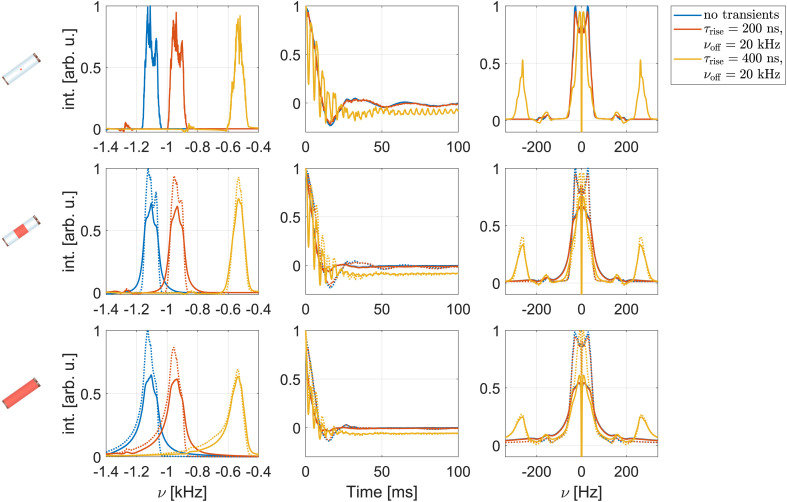
Simulated spectra (left) and echo dephasing curves (middle, with Fourier transform on the right) for a glycine methylene proton in a six-spin system with and without pulse transients under FSLG decoupling. The effective field along the magic angle was set to 125 kHz and a MAS frequency of 12.5 kHz assumed. Shown are simulations for the central volume element (top row, no rf-field inhomogeneity) as well as results taking the rf-field inhomogeneity for the central third (middle row) and the full sample space (bottom row) in a 1.9 mm probe into account. Dotted lines indicate simulations where only the static rf-field inhomogeneity was considered (C2). Solid lines indicate simulations taking the full rf inhomogeneity (radial and static, C1) into account. Pulse transients lead to a shifting of the non-refocused resonance and the appearance of an oscillating component in the dephasing curve.

Fig. 7 shows simulated FSLG-decoupled spectra and echo-dephasing curves of a methylene proton in glycine with and without pulse transients for an effective field of 125 kHz and a MAS frequency of 12.5 kHz. Model parameters *ν*_off_ and *τ*_rise_ (see eqn (23)) were chosen based on previous experimental measurements of pulse shapes on the proton channel.^42^ The changes of the FSLG pulse shape caused by the analytical transients are shown in Fig. S8 in the ESI.† In the simulated spectra, pulse transients lead to a shifting of the peak position as well as slight changes in the line shape. This change in peak position can be attributed to the imperfect compensation of the nutation in the transient-modified FSLG sequence and is also often observed experimentally. Overall, no significant influence on the line width can be seen either in the spectra or in the refocused lines.

However, an oscillating component arises in the simulated dephasing curves leading to sidebands in the Fourier transform for *τ*_rise_ = 400 ns. The amplitude of the oscillation is attenuated when the rf-field inhomogeneity is taken into account, but the sidebands remain visible even when the full sample space is considered. Such oscillations have also been observed experimentally^12^ and can be explained by a component of the effective field along the axis of the refocusing pulse.^43^ For *τ*_rise_ = 200 ns and a rf-field amplitude of 125 kHz, only marginal differences compared to simulations without pulse transients are observed. For higher rf-field amplitudes during FSLG, significantly stronger effects can be observed in simulations (see Fig. S9 in the ESI† for simulations at 250 kHz). Such oscillations can be significantly reduced in amplitude by using a double-echo experiment.^12,43^ The oscillating component in the dephasing curves makes fitting an exponential decay and thus the characterization of the refocused line width challenging (see Table S5 in the ESI† for resulting FWHM). Quantifying the broadening effects of pulse transients is therefore difficult. However, these results indicate that effects of pulse transients, similar to those of the radial rf-field inhomogeneity (see Fig. 6), are more pronounced at higher decoupling fields and can thus limit the achievable decoupling performance.

### 4.3 Line-width contributions by different orders of the effective Hamiltonian

In order to characterize the various terms contributing to the coherent line width under FSLG decoupling, simulations under effective Hamiltonians up to third order (see Section 2.1) were performed. Comparison with the results obtained from a time-slicing simulation of the full time-dependent Hamiltonian provides insight into the relative magnitude of the contribution to the line width and shape of the different orders. Moreover, this approach allows a further investigation of the origin of the observed line broadening with increasing effective field.

**Fig. 8:**
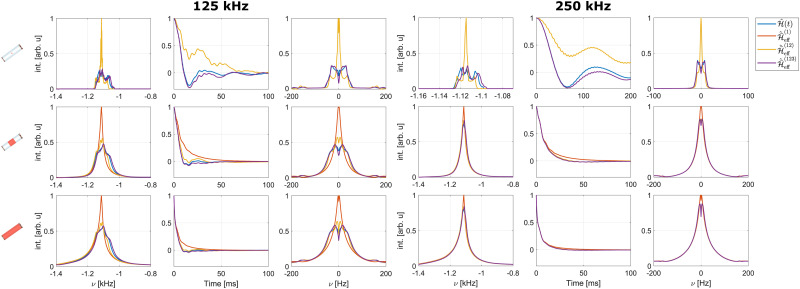
Simulated spectra and echo dephasing curves (and their Fourier transforms) under the full time-dependent Hamiltonian and effective Hamiltonians up to third order for one of the methylene protons in glycine under FSLG decoupling. Shown are simulation results for effective field strengths of 125 kHz (left panel) and 250 kHz (right panel). A MAS frequency of 12.5 kHz was assumed in both cases. Results are shown assuming a perfectly homogeneous rf field (top row) as well as taking the rf-field inhomogeneity including the radial contribution (C1) of the central third (middle row) and the full sample (bottom row) in a 1.9 mm rotor into account. In both simulated spectra and dephasing curves, third-order terms are necessary to approximate the full time-dependent Hamiltonian. Strong broadening is observed for the first-order effective Hamiltonian when the rf field inhomogeneity is taken into account. At an effective field strength of 250 kHz, the line shape and width, for the refocused as well as the non-refocused case, is dominated by the first order contribution when the rf-field inhomogeneity is taken into account (middle and bottom row).

Simulated spectra and echo dephasing curves of a methylene proton in glycine for the different 
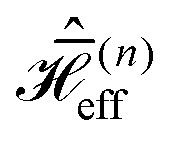
 are shown in Fig. 8 for effective field strengths of 125 kHz and 250 kHz. Since the first-order effective Hamiltonian for an ideal rf-field amplitude only contains the scaled isotropic chemical shift and would thus result in an infinitely sharp resonance, 
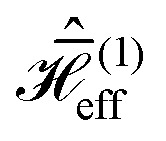
 is omitted for simulations without rf inhomogeneity (top row). Fig. 8 shows that the second-order effective Hamiltonians is insufficient to describe the line shape and width accurately for an effective field strength of 125 kHz (see Table S6 in the ESI† for FWHM) and third-order terms are necessary to approximate the time evolution observed under the full time-dependent Hamiltonian. Moreover, it can be seen that the rf inhomogeneity leads to a strong broadening in the first-order term. This has been shown to originate from the re-introduction of dipolar coupling terms in the first-order effective Hamiltonian due to periodic rf-field amplitude and phase modulations that arise from the radial contribution to the rf-field inhomogeneity^22^ and interfere with the first-order MAS averaging. This additional broadening is also present in Hahn echo simulations as the dipolar coupling cannot be refocused by a π pulse. At higher rf-field strengths, these first-order terms become the dominant contribution to the observed line width and severely limit the decoupling performance while at the same time the contributions by second- and third-order terms are strongly reduced. This explains the previous observation, that no further line narrowing can be achieved by increasing the rf field strength when the radial rf inhomogeneity is taken into account (see Fig. 6). Comparison of the resulting refocused and non-refocused line shapes (see Fig. S10 and S11 in the ESI†) shows that differences between the line shapes for the different orders of the effective Hamiltonian are negligibly small. This indicates that the various orders lead to a homogeneous broadening that cannot be refocused in a Hahn echo.

## 5. Conclusions

Using numerical simulations of effective Hamiltonians to different orders, we were able to disentangle the source of various contributions to the line width in FSLG decoupled spectra. In a full, unrestricted rotor, the main contribution to the residual line width stems from the rf-field inhomogeneity that leads to variations of the direction of the effective field. As a consequence of this, the projection of the isotropic chemical shift onto the effective field is different leading to a distribution of chemical shifts that manifest as a foot on the side of the line pointing away from the position of the rf-field carrier.^9,22^ This effect is strongest for parts of the rotor that experience the lowest rf-field amplitudes and can be strongly reduced by sample restriction using spacers or *B*_1_-field selective pulses in the rotating frame. Such a sample restriction leads to improved and more symmetric line shapes and the suppression of carrier-frequency artifacts.^11^ However, there are still significant differences between the line width in spectra and the refocused line width under Hahn-echo pulses even in such restricted samples. A large amount of the inhomogeneous broadening can be attributed to susceptibility broadening, sample inhomogeneity or imperfect shim while a smaller amount can be attributed to the remaining static rf-field distribution in the restricted sample.

The refocused line width has contributions from first-order, second-order, and third-order terms of the effective Hamiltonian. The first-order contributions are linked to the MAS-modulated rf-field inhomogeneity, while the second- and third-order contributions are generated by an incomplete averaging of the dipolar couplings at finite spinning frequencies. At moderate effective fields (around 100 kHz) all orders contribute significantly. Going to higher effective fields reduces the contributions of second- and third-order terms while the first-order terms become bigger and dominate the refocused line width. This leads to a plateau or even a broadening when increasing the strength of the effective field.

These observations have implications for the future design of novel pulse sequences to improve homonuclear decoupling. Overall, it appears to be crucial to improve the coil design such that the inhomogeneity of the rf field is reduced in the active sample volume. This encompasses both the improvement of the overall static rf homogeneity as well as reduction of the radial contribution to the rf inhomogeneity that causes the MAS modulation of the rf amplitude and phase. Moreover, it seems to be important to include the radial rf inhomogeneity into the design or numerical optimization process for the development of improved pulse sequences.^44^

## Conflicts of interest

There are no conflicts to declare.

## Supplementary Material

CP-025-D3CP00414G-s001
