# Supplementary material for: Residual proton line width under refocused frequency-switched Lee-Goldburg decoupling in MAS NMR
Source: Phys Chem Chem Phys. 2023 Mar 17;25(17):11959–70. doi: 10.1039/d3cp00414g (PMC10155489; doi:10.1039/d3cp00414g)
Supplement: CP-025-D3CP00414G-s001 [file CP-025-D3CP00414G-s001.pdf]

Electronic Supplementary Material (ESI) for Physical Chemistry Chemical Physics.
This journal is © the Owner Societies 2023

Supplementary Information:

Residual proton line width under refocused homonuclear frequency-switched Lee-Goldburg decoupling in MAS NMR

Kathrin Aebischer[a] and Matthias Ernst*[a]

[a]Physical Chemistry, ETH Zürich, Vladimir-Prelog-Weg 2, 8093 Zürich, Switzerland

*Corresponding author: maer@ethz.ch

Contents

S1 Additional Figures 2

S2 Additional Tables 11

S3 Pulse Programs 17

S3.1 Non-Refocused FWHM 17

S3.2 Refocused FWHM 19

S1 Additional Figures

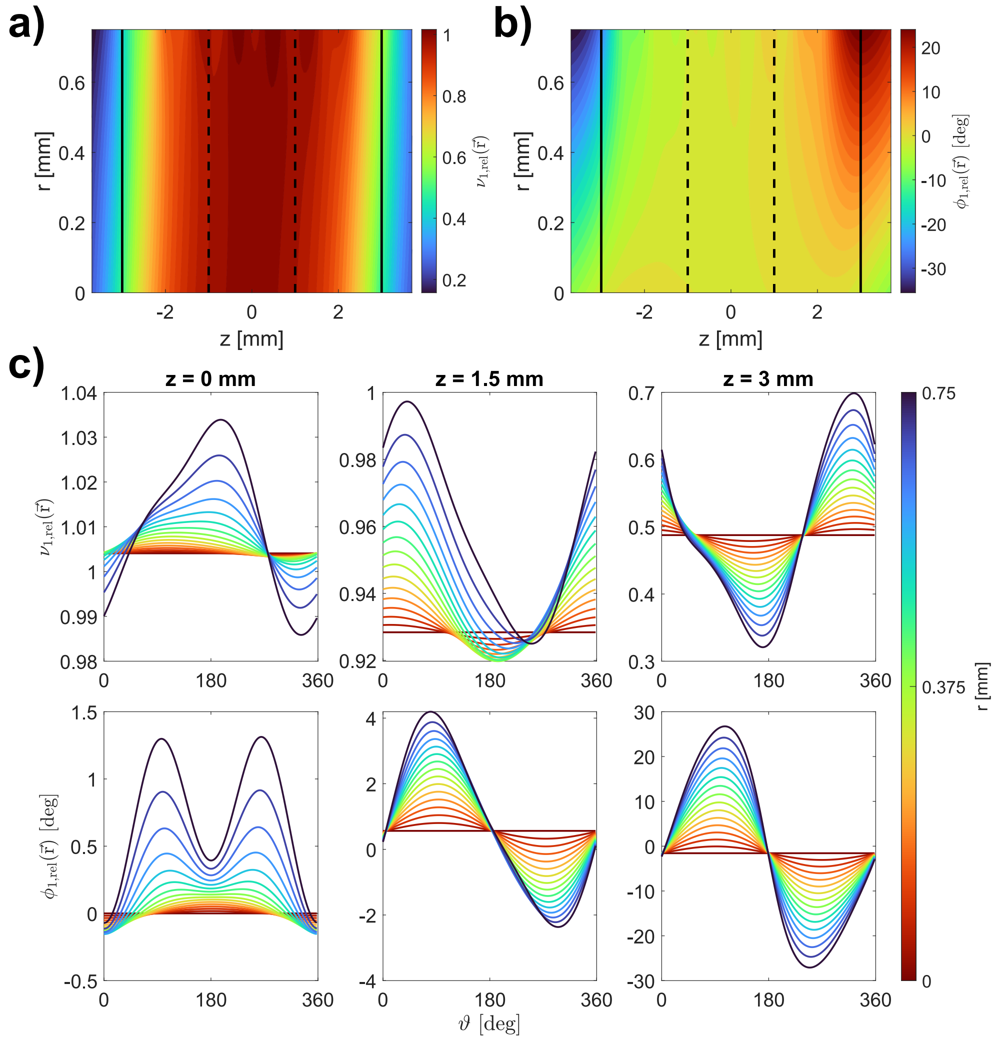

Figure S1: Simulated spatial radio-frequency field distribution in a 1.9 mm rotor. a) Relative rf-field amplitude $\nu_{1,\mathrm{rel}}$ as a function of r (radial distance from the rotor axis) and z (coordinate along the rotor axis) for $\vartheta = 90°$ (position of the rz-plane). For volume elements with $\nu_{1,\mathrm{rel}} = 1$, the rf-field amplitude experienced at this position corresponds to the nominal rf-field amplitude. b) Relative rf phase $\phi_{1,\mathrm{rel}}$ as a function of the position within the sample space, for an rz-plane with $\vartheta = 90°$. The phase is given relative to the phase experienced in the center of the rotor (at $r = z = 0$) In both a) and b) solid lines indicate the edge of the coil. For all simulations in this work, the sample space was restricted to the volume within the coil. Dashed lines indicate the central third of this sample space. c) Relative rf-field amplitude and phase as a function of the rotation angle ϑ at three different z-positions. When the sample is rotated during MAS ($\vartheta(t) = \omega_\mathrm{r} \cdot t$), spin packets experience rf-field amplitude and phase modulations that are periodic with the MAS frequency. The magnitude of these modulations increases towards the edges of the rotor.

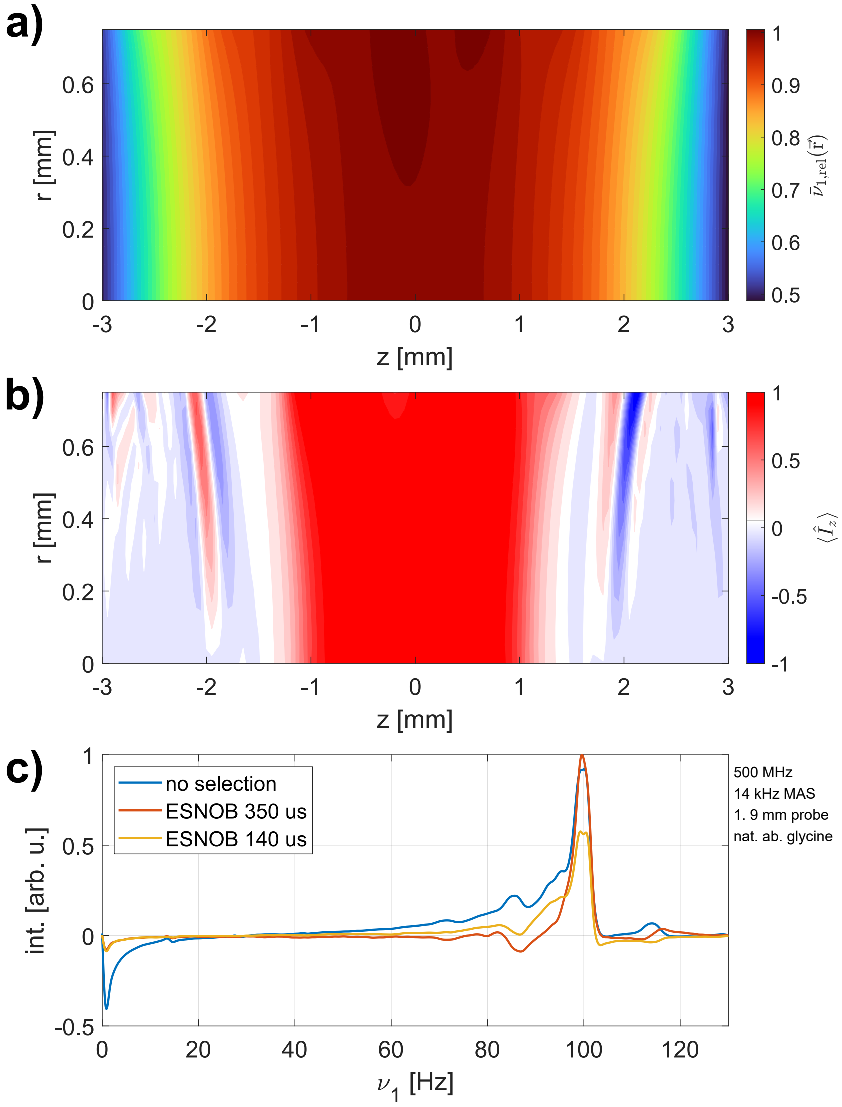

Figure S2: a) Spatial distribution of the mean value of the rf-field amplitude over a full rotor period $\bar{\nu}_{1,\mathrm{rel}}$ (sample space within the coil, r: radial distance, z: coordinate along the rotor axis). The rf-field amplitude drops off significantly along the rotor axis and the rf-field amplitude at the rotor edge is roughly half of the amplitude in the center. b) Simulated excitation profile of a nutation-frequency-selective e-SNOB pulse in the spinlock frame in the 1.9 mm rotor. The expectation value of $\hat{I}_z$ operator at the end of the selective pulse is shown as a function of the position within the rotor. A spinlock of 100 kHz was applied along x and the modulation frequency of the pulse set to 100 kHz. The density operator at the start of the pulse was set to $\hat{I}_x$. The pulse selectively excites parts of the sample, where the rf-field amplitude experienced corresponds to the nominal rf-field amplitude. For this e-SNOB pulse with a length of 350 μs, the excited region corresponds approximately to the central third of the sample space. The bandwidth can be adjusted by changing the length of the selective pulse. c) Experimental nutation spectra with and without a nutation-frequency-selective z-filter implemented prior to the nutation experiment recorded at 500 MHz proton Larmor frequency using a 1.9 mm probe. Spectra were measured at 14 kHz MAS using a sample of natural abundance glycine (fully packed). The nominal rf-field amplitude was calibrated to 100 kHz using a nutation spectrum. For the nutation-frequency-selective z-filter, the spinlock amplitude and the modulation frequency of the selective pulse were set to 100 kHz. This shows, that we can indeed selectively excite part of the rf distribution using such pulses. More details on nutation-frequency-selective pulses and their implementation can be found in Aebischer et al. Magnetic Resonance, 1, 187-195 (2020).

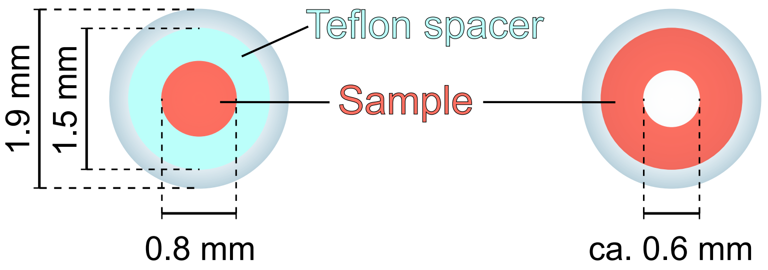

Figure S3: Schematic depiction of the sample space distribution in the loosely packed (right) and the radially restricted 1.9 mm rotor (left). Sample restriction in the radial dimension was achieved by inserting a cylindrical tube-shaped Teflon spacer in the rotor with central hole with a diameter of 0.8 mm. The powdered sample was then packed into this central hole. The loosely packed rotor was spun at 30 kHz for 48 h. The spinning leads to the packing of the powdered sample close to the rotor walls. Observation of the sample distribution under a microscope after spinning revealed that a hole without sample (diameter of ca. 0.6 mm) resulted in the middle. Such a hole is also observed in the fully packed sample, however its diameter is significantly smaller (< 0.3 mm).

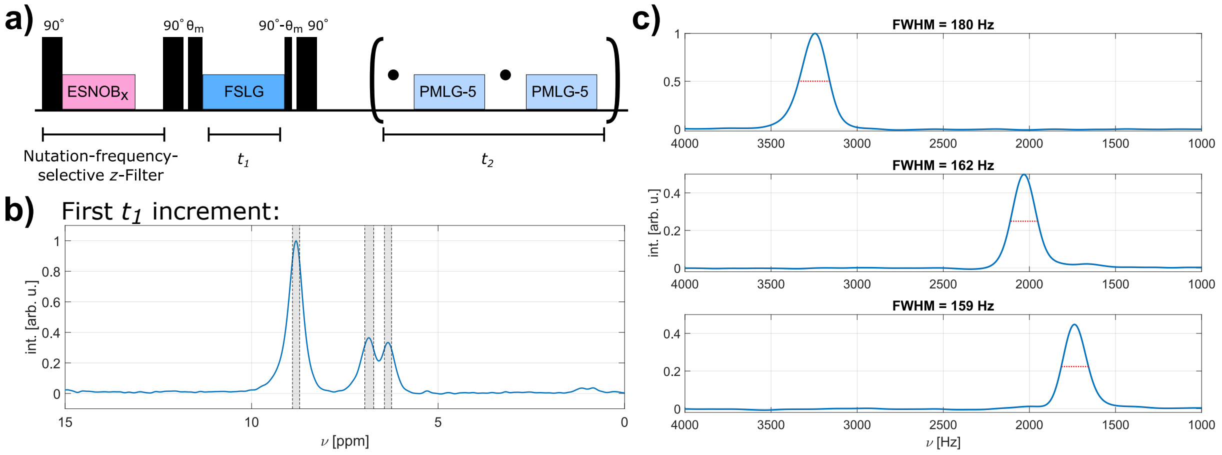

Figure S4: Example of the implemented data processing for measurements of non-refocused proton FWHM. Data shown was recorded at 500 MHz proton Larmor frequency using a 1.9 mm Bruker probe at 14 kHz MAS and a sample of natural abundance glycine (fully packed rotor). a) Schematic of the pulse sequence used to measure 2D 1H-1H chemical shift correlation spectra with FSLG decoupling (effective field of 125 kHz along magic angle for the data shown here) in the indirect dimension. Chemical shift resolution in the direct dimension is achieved using wPMLG. b) Resulting spectrum in the direct dimension for the first t_1 increment. This spectrum is used to define the summation ranges in ω_2 for the three separate peaks. The summation ranges are set to $0.5 \cdot \mathrm{FWHM}_{t_2}$ for each peak (shaded areas). c) The non-refocused FWHM of each peak are obtained from the Fourier transform of the summed ranges. No chemical shift scaling is applied in either dimension.

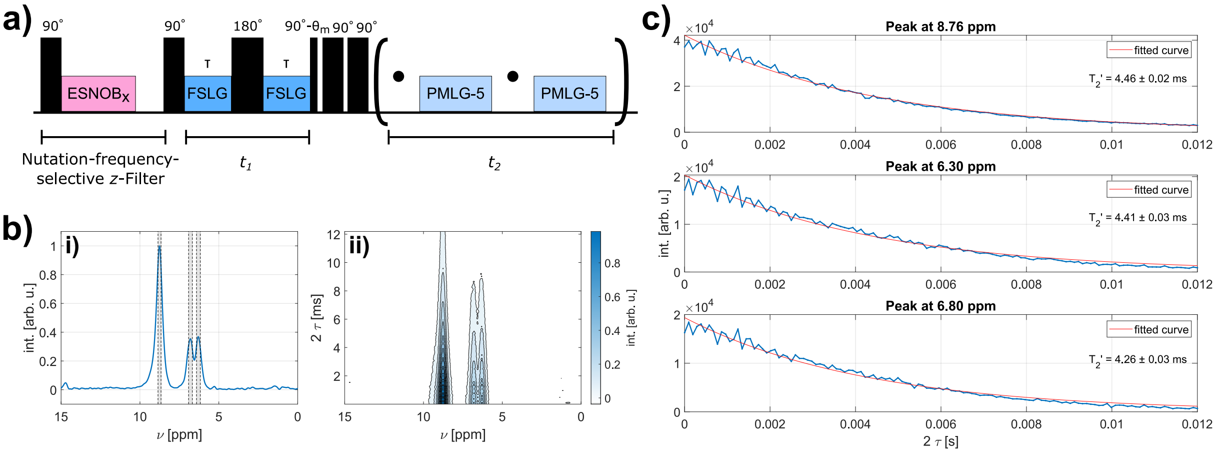

Figure S5: Example of the implemented data processing for measurements of refocused proton FWHM. Data shown was recorded at 500 MHz proton Larmor frequency using a 1.9 mm Bruker probe at 14 kHz MAS and a sample of natural abundance glycine (fully packed rotor). a) Schematic of the pulse sequence used to measure T_2' under FSLG decoupling (effective field of 125 kHz along the magic angle for the data shown here) in pseudo-2D proton experiments. Chemical shift resolution in the direct dimension is achieved using wPMLG. b) i) Resulting spectrum in the direct dimension for the first t_1 increment. This spectrum is used to define the integration ranges in ω_2 for the three separate peaks. The integration ranges are set to $0.5 \cdot \mathrm{FWHM}_{t_2}$ for each peak (shaded areas). ii) Contour plot of the intensity as a function of the echo time τ in the indirect dimension. c) Dephasing curves (integrated intensity as a function of the echo time τ) for each of the three glycine proton resonances. Fitting the data with an exponential decay allows the determination of the dephasing time T_2'. The refocused FWHM can then be calculated as $\mathrm{FWHM} = \frac{1}{\pi T_2'}$.

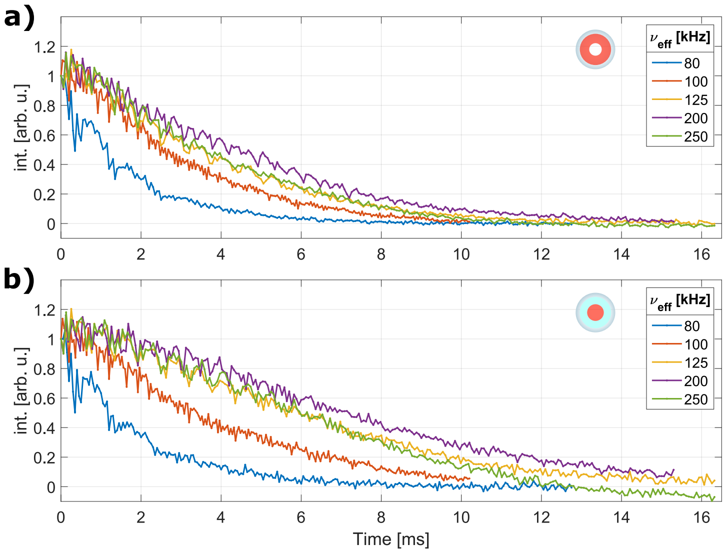

Figure S6: Experimental echo dephasing curves of one of the two methylene protons in glycine (natural abundance) measured at 500 MHz proton Larmor frequency at a spinning frequency of 14 kHz for different effective field strengths during the FSLG decoupling in the indirect dimension. A nutation-frequency-selective z-filter (100 kHz spinlock, 350 μs e-SNOB pulse with 100 kHz modulation) was used to reduce the static rf-field inhomogeneity. Results are shown for a) loosely packed sample and b) the radially restricted sample. The sample space distribution is indicated in red in the rotor schematics shown in the top right-hand corner in each plot.

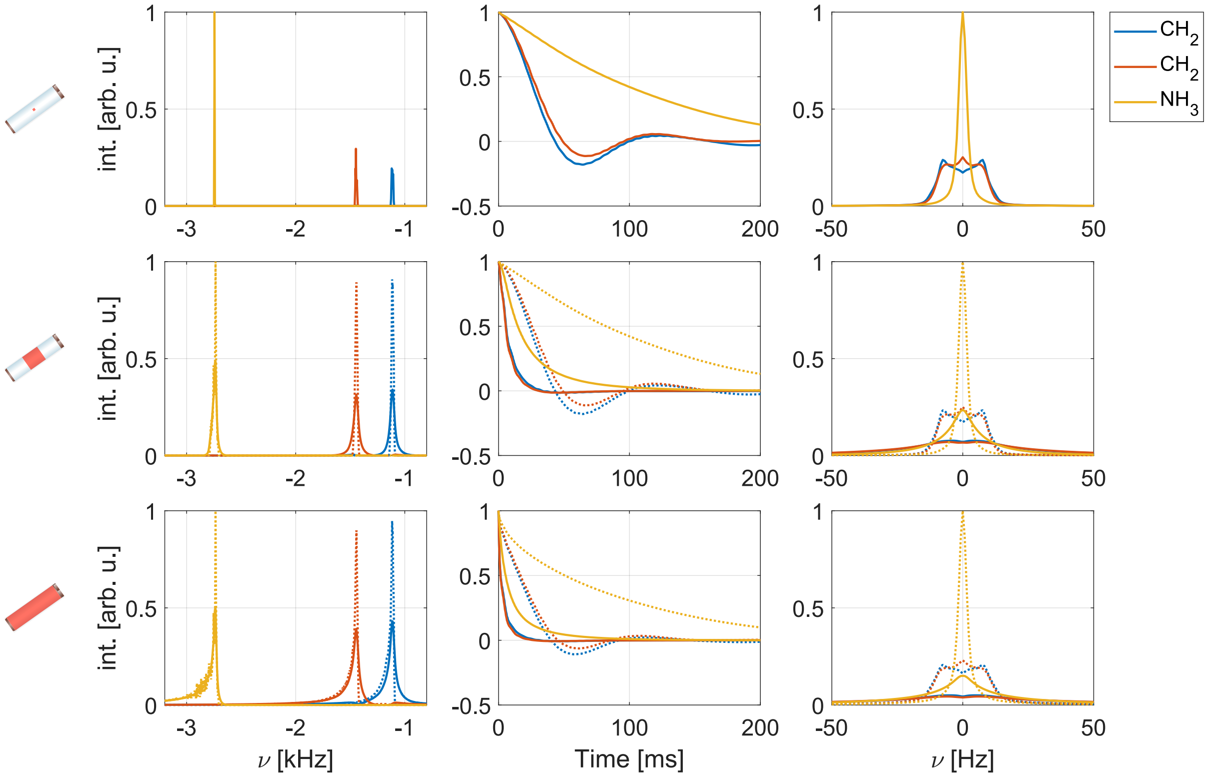

Figure S7: Simulated spectra (left) and dephasing curves (middle as well as their Fourier transform on the right) for the three central protons in a six-spin system based on glycine under FSLG decoupling (single-spin detection). The effective field strength was set to 250 kHz and a spinning speed of 12.5 kHz assumed. Shown are simulations for the central volume element (no rf-field inhomogeneity, top row) as well as the central third (middle row) and the full sample space of a 1.9 mm rotor (bottom row). Solid lines correspond to C1 (both static and radial rf-field inhomogeneity taken into account, s. Section 3.1 in the main text), dotted lines to C2 (only static rf-field inhomogeneity). Modulations of the rf-field amplitude and phase due to the radial rf-field inhomogeneity lead to line broadening and shortened dephasing times in echo simulations. In comparison to the results at a decoupling field of 125 kHz shown in the main text (s. Fig. 4), these broadening effects are stronger for higher rf fields and the radial rf-field inhomogeneity thus severely limits the decoupling performance.

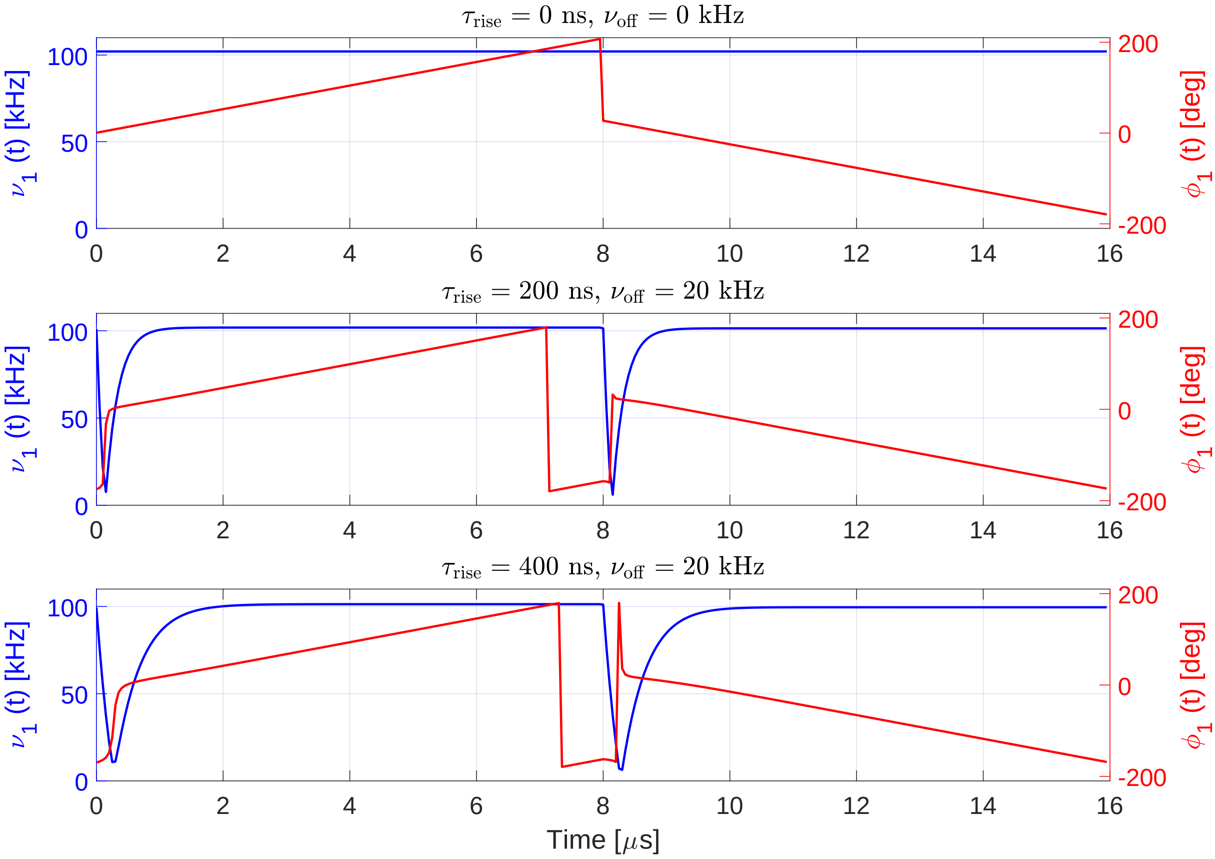

Figure S8: Radiofrequency field amplitude and phase as a function of time for a phase ramp corresponding to FSLG decoupling with an effective field strength of 125 kHz along the magic angle. The top panel shows the ideal phase ramp without pulse transients. The bottom two panels correspond to cases where pulse transients with $\tau_{\rm rise} = 200$ ns (middle) and 400 ns (bottom) with $\nu_{\rm off} = 20$ kHz were taken into account based on the simple model presented in the main text (s. Eq. (23)). The 180° phase jumps introduce distortions of both the rf amplitude and rf phase. These rf shape files were used as input in numerical simulations to study the effect of pulse transients on the refocused and non-refocused line widths.

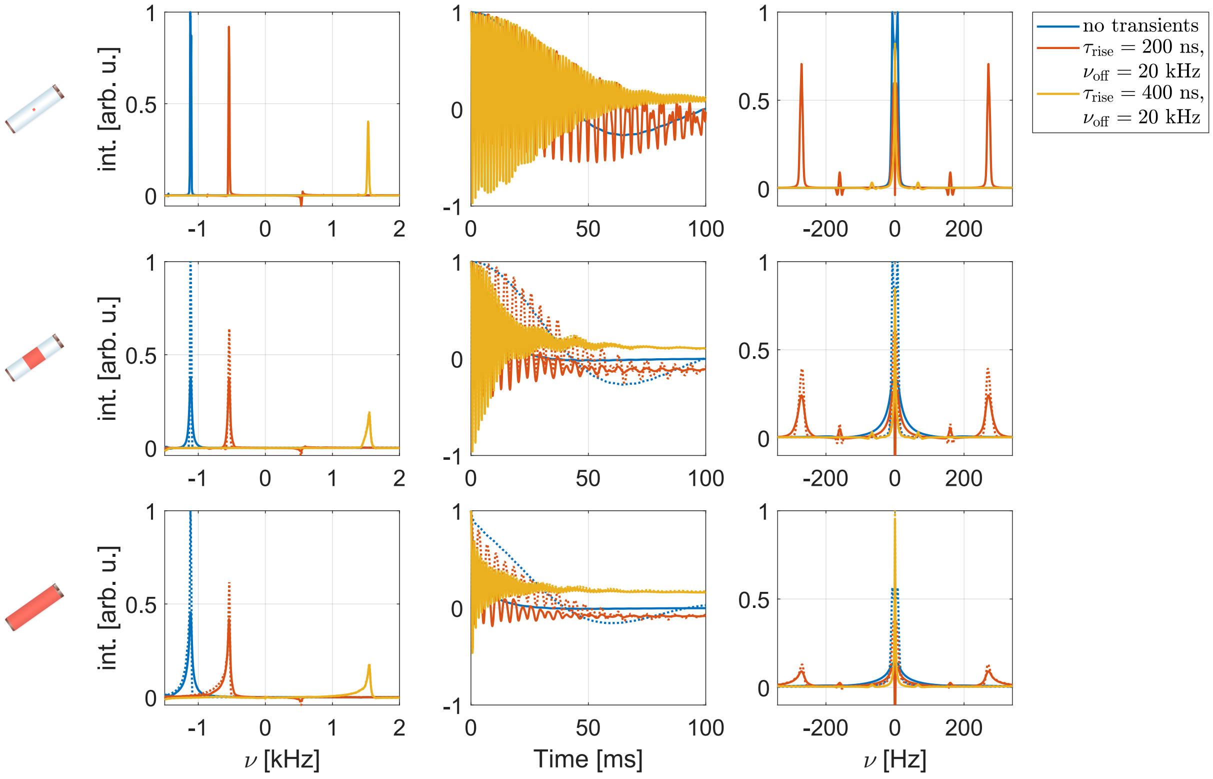

Figure S9: Simulated spectra (left) and echo dephasing curves (middle, with Fourier transform on the right) for a glycine methylene proton in a six-spin system with and without pulse transients under FSLG decoupling. The effective field along the magic angle was set to 250 kHz and a MAS frequency of 12.5 kHz was assumed. Shown are simulations for the central volume element (top row, no rf-field inhomogeneity) as well as results taking the rf-field inhomogeneity for the central third (middle row) and the full sample space (bottom row) in a 1.9 mm probe into account. Dotted lines indicate simulations where only the static rf-field inhomogeneity was considered (C2). Solid lines correspond to simulations taking the radial rf inhomogeneity into account in addition (C1). Pulse transients lead to a shifting of the non-refocused resonance and the appearance of an oscillating component in the dephasing curve. Compared to the results for a decoupling field strength of 125 kHz shown in the main text (s. Fig. 7), the amplitude of the oscillation is significantly stronger, making an exponential fit of the dephasing curve difficult.

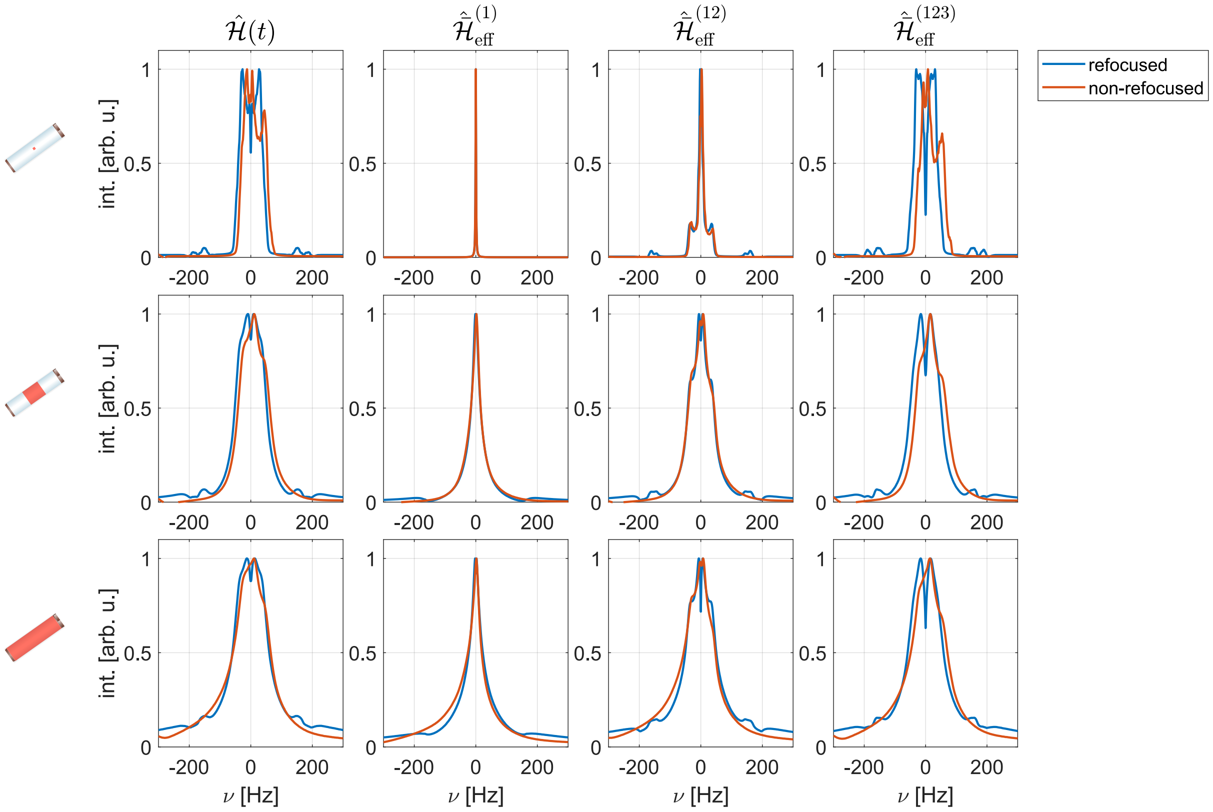

Figure S10: Comparison of the resulting refocused and non-refocused line shapes of one of the methylene protons in glycine (six-spin system) under homonuclear FSLG decoupling for simulations of different orders of effective Hamiltonians $\hat{\bar{\mathcal{H}}}_{\text{eff}}^{(n)}$. Results for a time-slicing simulation of the full time-dependent Hamiltonian $\hat{\mathcal{H}}(t)$ are shown for comparison in the first column. The effective field strength was set to 125 kHz and a MAS frequency of 12.5 kHz assumed. All spectra were processed with 2 Hz exponential line broadening and non-refocused lines shifted by the scaled isotropic chemical shift. Simulations in the top row assumed an ideal rf field, while simulations in the middle and bottom row took the rf-field inhomogeneity (including the radial contribution, C1) in the central third and the full sample space of a 1.9 mm rotor into account. The resulting refocused and non-refocused line shapes are very similar for the different orders of the effective Hamiltonian. This shows that their contribution to the line width is homogeneous and can therefore not be refocused by a Hahn echo. Only the distribution of chemical shift scaling factors due to the static rf inhomogeneity is refocused in an echo experiment (removes asymmetric feet of lines, most pronounced in the full sample space).

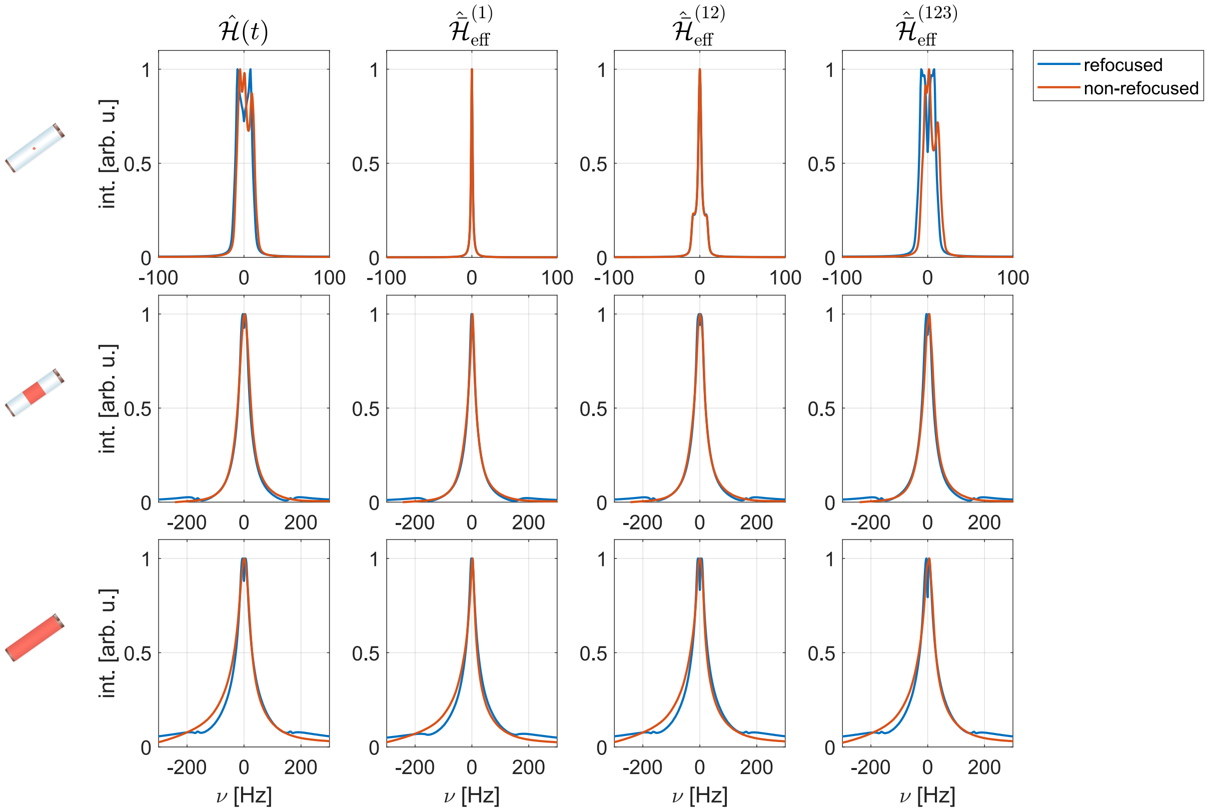

Figure S11: Comparison of the resulting refocused and non-refocused line shapes of one of the methylene protons in glycine (six-spin system) under homonuclear FSLG decoupling. The effective field strength was set to 250 kHz and a MAS frequency of 12.5 kHz assumed. All spectra were processed with 2 Hz exponential line broadening. Simulations in the top row assumed an ideal rf field, while simulations in the middle and bottom row took the rf-field inhomogeneity (including the radial contribution, C1) in the central third and the full sample space of a 1.9 mm rotor into account. Non-refocused lines were shifted by the scaled isotropic chemical shift. Note the different scaling of the x-axis for simulations in the top row. The refocusing behaviour of the different order contribution is similar to what was observed for simulations at a lower decoupling field strength of 125 kHz (s. Fig. S10), but the line width is dominated by the first-order contribution for higher effective field strengths.

S2 Additional Tables

Table S1: Comparison of experimental and simulated proton FWHM (non-refocused as well as refocused) for glycine under homonuclear FSLG proton decoupling with an effective field strength of 125 kHz in a 1.9 mm rotor.

Non-refocused FWHM [Hz]

Spin	Experiment[1,2]	Simulation				
		Central vol. element[3]	Restricted sample		Full sample	
			C1	C2	C1	C2
CH_2	162	85	102	86	120	96
CH_2	159	41	95	71	111	83
NH_3	180	14	37	34	42	35

Refocused FWHM [Hz]

Spin	Experiment[1]	Simulation[4]				
		Central vol. element	Restricted sample		Full sample	
			C1	C2	C1	C2
CH_2	76	55	71	55	123	70
CH_2	78	46	66	46	124	60
NH_3	75	8	16	8	34	9

[1] Fully packed rotor, 14 kHz MAS, 350 μs *e*-SNOB pulse for the nutation-frequency-selective *z*-filter.

[2] Experimental FWHM are taken from processed spectra without chemical shift scaling.

[3] Spectra processed with 2 Hz exponential line broadening.

[4] Obtained from exponential fit of the simulated dephasing curve.

Table S2: Comparison of experimental and simulated proton FWHM (non-refocused as well as refocused) for glycine under homonuclear FSLG proton decoupling with an effective field strength of 250 kHz in a 1.9 mm rotor.

Non-refocused FWHM [Hz]

Spin	Experiment[1,2]	Simulation				
		Central vol. element[3]	Restricted sample		Full sample	
			C1	C2	C1	C2
CH_2	189	21	46	24	60	28
CH_2	180	19	50	23	65	26
NH_3	177	4	36	9	41	9

Refocused FWHM [Hz]

Spin	Experiment[1]	Simulation[4]				
		Central vol. element	Restricted sample		Full sample	
			C1	C2	C1	C2
CH_2	75	22	43	21	91	27
CH_2	75	19	45	19	101	24
NH_3	70	2	13	2	30	3

[1] Fully packed rotor, 14 kHz MAS, 350 μs *e*-SNOB pulse for the nutation-frequency-selective *z*-filter.
[2] Experimental FWHM are taken from processed spectra without chemical shift scaling.
[3] Spectra processed with 2 Hz exponential line broadening.
[4] Obtained from exponential fit of the simulated dephasing curve.

Table S3: Simulated refocused and non-refocused FWHM for glycine under homonuclear FSLG proton decoupling with an effective field strength of 125 kHz in a 1.9 mm rotor. A spinning speed of 12.5 kHz was assumed. FWHM are given for C1 (static and radial rf-field inhomogeneity) and C2 (static rf-field inhomogeneity only). Simulation results of individual volume elements were weighted with the simulated excitation efficiency of a 350 μs *e*-SNOB pulse in the spinlock frame (s. Fig. S2b in the SI). The obtained line widths are very similar to the ones obtained for the restriction of the sample space to the central third presented in the main text (s. Table S1).

Spin	Non-refocused FWHM [Hz]		Refocused FWHM [Hz][1]	
	C1	C2	C1	C2
CH_2	104	88	76	57
CH_2	97	72	72	47
NH_3	39	33	18	8

[1] Obtained from exponential fit of the simulated dephasing curve.

Table S4: Simulated proton FWHM (non-refocused as well as refocused) for glycine under homonuclear FSLG proton decoupling with an effective field strength of 125 kHz in a 1.9 mm rotor for simulations of an eight-spin system (instead of the six-spin system results shown in Table S1). Simulations took the full rf-field inhomogeneity including the radial contribution into account (C1). Compared to the results from six-spin simulations, the additional spins lead to approximately 10 Hz broadening for the methylene protons.

Simulated Non-refocused FWHM [Hz]

Spin	Central vol. element[1]	Restricted sample	Full sample
CH_2	91	110	133
CH_2	69	107	127
NH_3	32	72	106

Simulated Refocused FWHM [Hz][2]

Spin	Central vol. element	Restricted sample	Full sample
CH_2	63	78	136
CH_2	56	76	138
NH_3	29	49	88

[1] Spectra processed with 2 Hz exponential line broadening.

[2] Obtained from exponential fit of the simulated dephasing curve.

Table S5: Summary of simulated refocused and non-refocused FWHM of a glycine methylene proton under FSLG decoupling with and without pulse transients in a 1.9 mm probe. Due to the strong oscillating component in the dephasing curves for an effective field strength of 250 kHz, characterizing the refocused FWHM is challenging and an exponentially decaying oscillation $((1-a)\cos(\omega t)\exp(-t/T_2')+a)$ was fit as well as a simple exponential decay. The two fits lead to significant differences in the obtained T_2', making the characterization of the refocused FWHM challenging.

Non-refocused FWHM [Hz]

$\nu_{\mathrm{eff}}^{\mathrm{FSLG}}$ [kHz]	τ_{rise} [ns]	ν_{off} [kHz]	Central vol. element[1]	Restricted sample C1	Restricted sample C2	Full sample C1	Full sample C2
125	0	0	85	102	86	120	95
	200	20	86	104	89	124	104
	400	20	64	87	73	106	97
250	0	0	21	46	24	60	29
	200	20	18	41	28	52	30
	400	20	26	58	61	59	61

Refocused FWHM [Hz][2]

$\nu_{\mathrm{eff}}^{\mathrm{FSLG}}$ [kHz]	τ_{rise} [ns]	ν_{off} [kHz]	Central vol. element	Restricted sample C1	Restricted sample C2	Full sample C1	Full sample C2
125	0	0	55	71	55	123	70
	200	20	54	71	54	124	70
	400	20	47	64	48	126	66
250	0	0	14	38	14	86	18
	200	20	15	45	15	150[3]	22
	400	20	1629 (8)[4]	1643 (23)[4]	1627 (22)[4]	1532 (74)[4]	1375 (71)[4]

[1] Spectra processed with 2 Hz exponential line broadening.

[2] Obtained from exponential fit of the simulated dephasing curve.

[3] Oscillations in dephasing curve distort fit, the second CH_2 proton is less affected.

[4] Fitted with decaying oscillation instead of exponential decay.

Table S6: Simulated FWHM for different orders of the effective Hamiltonian in comparison to the full time-dependent Hamiltonian $\hat{\mathcal{H}}(t)$ under homonuclear FSLG decoupling with an effective field strength of 125 kHz in a 1.9 mm rotor.

Non-refocused FWHM [Hz]

Spin	Central vol. element[1]			Restricted sample				Full sample			
	$\hat{\mathcal{H}}(t)$	$\hat{\mathcal{H}}_{\text{eff}}^{(12)}$	$\hat{\mathcal{H}}_{\text{eff}}^{(123)}$	$\hat{\mathcal{H}}(t)$	$\hat{\mathcal{H}}_{\text{eff}}^{(1)}$	$\hat{\mathcal{H}}_{\text{eff}}^{(12)}$	$\hat{\mathcal{H}}_{\text{eff}}^{(123)}$	$\hat{\mathcal{H}}(t)$	$\hat{\mathcal{H}}_{\text{eff}}^{(1)}$	$\hat{\mathcal{H}}_{\text{eff}}^{(12)}$	$\hat{\mathcal{H}}_{\text{eff}}^{(123)}$
CH_2	85	13	80	102	28	83	100	120	38	99	116
CH_2	41	8	35	95	35	69	92	111	48	86	108
NH_3	14	5	15	37	18	30	34	42	19	34	39

Refocused FWHM [Hz][2]

Spin	Central vol. element			Restricted sample				Full sample			
	$\hat{\mathcal{H}}(t)$	$\hat{\mathcal{H}}_{\text{eff}}^{(12)}$	$\hat{\mathcal{H}}_{\text{eff}}^{(123)}$	$\hat{\mathcal{H}}(t)$	$\hat{\mathcal{H}}_{\text{eff}}^{(1)}$	$\hat{\mathcal{H}}_{\text{eff}}^{(12)}$	$\hat{\mathcal{H}}_{\text{eff}}^{(123)}$	$\hat{\mathcal{H}}(t)$	$\hat{\mathcal{H}}_{\text{eff}}^{(1)}$	$\hat{\mathcal{H}}_{\text{eff}}^{(12)}$	$\hat{\mathcal{H}}_{\text{eff}}^{(123)}$
CH_2	55	14	59	71	33	59	72	123	85	116	122
CH_2	46	17	48	66	39	55	66	124	99	116	122
NH_3	8	7	8	16	12	15	16	33	30	33	33

[1] Spectra processed with 2 Hz exponential line broadening.

[2] Obtained from exponential fit of the simulated dephasing curve.

Table S7: CSA tensors for the homonuclear proton spin system used in simulations. For the spin system consisting of six protons, spins number 4 and 5 are omitted. Parameters are given in the principal axis system (PAS) of the tensors at 600 MHz proton larmor frequency. Euler angles α, β and γ, given in degree, describe transformation from PAS to the lab frame (given in ZYZ-convention).

Spin	ν_{iso} [Hz]	δ_{CSA} [Hz]	η_{CSA}	α	β	γ
1 (Hα1)	1932	2100	0.8013	-54.76	109.2	96.18
2 (Hα2)	2508	-2974.8	0.751	-17.82	103.4	78.42
3 (HN)	4752	2085.6	0.998	-2.092	88.39	69.72
4 (HN)	4752	2085.6	0.998	-2.092	88.39	69.72
5 (HN)	4752	2085.6	0.998	-2.092	88.39	69.72
6 (Hα2)	2508	-2974.8	0.751	-17.82	103.4	78.42
7 (Hα2)	2508	-2974.8	0.751	-17.82	103.4	78.42
8 (Hα1)	1932	2100	0.8013	-54.76	109.2	96.18

Table S8: Dipolar coupling tensors for the homonuclear proton spin system used in simulations (given is the anisotropy of the dipolar coupling tensor). For the spin system consisting of six protons, spins number 4 and 5 are omitted. Scalar J-couplings were neglected in simulations (set to 0 Hz). Euler angles α, β and γ, given in degree, describe transformation from PAS to the lab frame (given in ZYZ-convention).

Spin pair	δ_{DD} [Hz]	α	β	γ
(1,2)	-52245	0	70.7704	120.471
(1,3)	-13595	134.371	96.5903	14.8764
(1,4)	-13595	134.371	96.5903	14.8764
(1,5)	-11213	134.371	96.5903	14.8764
(1,6)	-2983.4	0	42.7559	143.03
(1,7)	-11683	0	135.41	69.0396
(1,8)	-4926.5	0	113.094	234.532
(2,3)	-13387	-105.815	85.9751	47.2049
(2,4)	-13387	-105.815	85.9751	47.2049
(2,5)	-11041	-105.815	85.9751	47.2049
(2,6)	-9027.6	0	31.8856	167.065
(2,7)	-9027.6	0	148.114	12.9347
(2,8)	-2213.4	0	115.015	255.244
(3,4)	-30665	0	81.8395	28.4886
(3,5)	-24851	0	81.8395	28.4886
(3,6)	-6475.4	-124.44	138.512	-96.44
(3,7)	-2553.1	-53.6713	118.981	35.751
(3,8)	-8747	79.9454	130.848	-83.2699
(4,5)	-23441	0	81.8395	28.4886
(4,6)	-6475	-124.44	138.512	-96.44
(4,7)	-2553.1	-53.6713	118.981	35.751
(4,8)	-8747	79.9454	130.848	-83.2699
(5,7)	-2553.1	-53.6713	118.981	35.751
(5,8)	-8747	79.9454	130.848	-83.2699
(6,7)	-1152.5	0	148.771	0
(6,8)	-899.91	0	134.988	275.494
(7,8)	-1641.1	0	84.3594	239.803

S3 Pulse Programs

S3.1 Non-Refocused FWHM

```
;wPMLG2D_B1zFilter_theta_clean.kaab
;K. Aebischer, 29.09.2021
;based on: wpmlg2d (TOPSPIN 3.0)
;proton-proton spin diffusion 2D experiment with decoupling in t1
;and wPMLG detection in t2
;nutation-frequency-selective z-Filter to reduce rf inhomogeneity:
;Aebischer et al. MR 1, p. 523-543, (2021)
;digital mode
;in t2: wPMLG, purified version: Leskes, Madhu and Vega, Chem. Phys. Lett. 447,370 (2007)
;using shape files for decoupling in t1 and wPMLG in t2
;eg. spnam1 = m5m or m3m, m5p or m3p (Bruker shapes)
;different carrier positions in t1 and t2
;AV III instruments only!!
;requires dccorr off to be typed for shorter delays
;NS = N*8 to cancel artefacts
;FnMODE: STATES-TPPI to be able to correct for peak folding
;Set:
;PULSES:
;p1  :  proton 90 degree pulse at pl12
;p10 :  length of decoupling shapefile in t1
;p15 : selective excitation in z-Filter
;p5  :  PMLG cycle/2
;
;POWER LEVELS:
;plw1  : proton 90d pulse
;plw12 :  power level for initial excitation pulses
;plw13 : power level for PMLG in t2 (for simplicity: plw13 = plw12)
;
;DELAYS:
;d1  :  recycle delay
;d2  : dephasing delay for z-Filter
;d21 :  delay after RG_OFF (eg 0.3 us)
;d22 :  delay before RG_ON (deadtime, eg. 3.4 us)
;d25 :  theta1 in s = deg, orientation of eff. field for FSLG in t1
;
;CONSTANTS:
;cnst10 :  carrier offset for FSLG in t1 in Hz
;cnst11 :  carrier offset for wPMLG in t2 in Hz
;cnst20 :  rf field strength along thetaM for wPMLG in t2 (used to compute P5)
;
;COUNTER
;l11 : no. of oversampled datapoints in t2 (eg 6)
;$COMMENT=homonuclear decoupling with w-pmlg
;$CLASS=Solids
;$DIM=2D
;$TYPE=homonuclear decoupling
;$SUBTYPE=explicit acquisition
;$OWNER=Bruker
"acqt0=0"
"spw1=plw13" ;power level for PMLG in t2
dwellmode auto
#include <Avancesolids.incl>
#include <Delayssolids.incl>
```

```
#include <lgcalc.incl>
/*;set cnst20 to RF field at pl13, wpmlg pulse calculated as multiple of 100 ns */
"d9 = 0.1u*(l11)" ;set the sampling window in Avancedru.incl
;set initial value for d0 (t1 loop counter)
"d0 = 1.0u"
"l0 = 0"
;Pulses for FSLG decoupling in t1
"p3 = (d25/90)*p1" ;theta1 = orientation of eff. field during t1
"p2 = p1-p3" ;90d - theta1
;default delays
  "d60=0.1u"
  "d62=1.0u"
define delay dead
"dead = d22" ;delay before RG_ON (deadtime)
"p9 = dead+0.1u+d21+d9" ;total length of window in t2
define pulse smooth
define loopcounter polish
define pulse wpmlg
"smooth = 10*p5"  ;p5 computed from cnst20 (approx. rf power during PMLG)
"polish = 1u*smooth/10"
"wpmlg = 2u*polish" ;length of wPMLG shape in t2
define delay cycle
"cycle = p9+wpmlg" ;cycle time for wPMLG
define loopcounter count ;loop counter for data acquisition t2
define loopcounter nrows
"nrows = td1/2"
"count = aq/2*cycle-0.5" ;make sure td datapoints sampled in t2 (2 per loop)
define delay rest ;make sure sampling proceeds throughout the sequence
   "rest = aq-(2*count*cycle)" ;2*count*cycle is total actual acquisition time
   "blktr2 = 0.7u" ;opens transmitter gate 0.7 usec before pulse
;(transmitter noise not sampled)
;Compute spectral width that should be set
define loopcounter setswh
"setswh = 1/cycle" ;set swh2 to this value
define delay setdwell
"setdwell = 2/setswh"
; #----- START OF PULPROG
#include <fastCRAMPS.incl>
1 ze ;acquire into a cleared memory
   setdwell ;show calculated dwell and swh in F1
2 50m do:f1
  .1u fq=cnst10 :f1  ;set carrier for t1
  10u reset:f1
; #--- Saturation
3 1u pl12:f1
  (p1 pl12 ph30):f1
  25m
  lo to 3 times 5
4 d1 pl12:f1        ;recycle delay
  "l0=d0/p10" ;increment l0 (t1)
; #--- Nutation-frequency-selective z-filter 1H
  (p1 pl12 ph1):f1 ;90d hard excitation
  (p15:sp15 ph21):f1 ;selective excitation (shaped pulse)
  d2 ;dephasing delay
; #--- Decoupling in t1
  (p1 pl12 ph0):f1 ;90d (states)
  (p3 pl12 ph6):f1 ;theta1
```

```
  d60 pl20:f1
  d62
5  (p10:spf10 ph11):f1 ;FSLG (shaped pulse)
  lo to 5 times l0 ;repeat l0 time
; #--- wPMLG detection in t2
  d62 pl12:f1
  STARTADC ;prepare adc for sampling, set reference frequency, defined in Avancedru.incl
  RESETPHASE ;reset reference phase
  (p2 pl12 ph6):f1 ;90d - theta1
  .1u fq=cnst11 :f1  ;set carrier for wPMLG (t2)
  1u
  (p1 pl12 ph7):f1
  .1u DWL_CLK_ON
6 dead  pl13:f1
  d9 RG_ON ;receiver gain on for d9
  .1u RG_OFF             ;take l11  complex data points
  d21
  (wpmlg:sp1  ph10^):f1 ;wPMLG, phase = x
  dead
  d9 RG_ON  ;receiver gain on for d9
  .1u RG_OFF ;take l11  complex data points
  d21
  (wpmlg:sp1  ph10^):f1 ;wPMLG, phase = -x
  lo to 6 times count ;make sure td points are sampled
  rest
  1u  DWL_CLK_OFF
  rcyc=2  ;next scan
7 100m wr #0 if #0 zd ;save data
  1m ip0  ;increments ph0 by 90 deg
  lo to 2 times 2   ;states phase cycling
  .1u rp0  ;reset phase for states
8 1m id0 ;increment loop counters
  1m
  lo to 2 times nrows
  exit ;finished
; #--- Phase cycles
ph0  = 0 ;excitation in t1
ph1  = 3 1 ;excitation in z-Filter
ph21 = 0 ;selective excitation in z-Filter
ph6  = 1 ;theta1 and 90d - theta1
ph7  = 0 0 1 1 2 2 3 3 ;90d for wPMLG detection
ph10 = 0 2 ;wPMLG in t2
ph11 = 0 ;FSLG in t1
ph30 = 0 ;saturation pulses
ph31 = 0 2 1 3 2 0 3 1 ;receiver
```

S3.2 Refocused FWHM

```
;wPMLG2D_1E_B1zFilter_theta_clean.kaab
;K. Aebischer, 29.09.2021
;based on: wpmlg2d (TOPSPIN 3.0)
;proton-proton spin diffusion 2D experiment with decoupling in t1
;and wPMLG detection in t2
;refocusing pulse in t1 (measurement of T2p)
;nutation-frequency-selective z-Filter to reduce rf inhomogeneity:
;Aebischer et al. MR 1, p. 523-543, (2021)
;digital mode
```

```
;in t2: wPMLG, purified version: Leskes, Madhu and Vega, Chem. Phys. Lett. 447,370 (2007)
;using shape files for decoupling in t1 and wPMLG in t2
;eg. spnam1 = m5m or m3m, m5p or m3p (Bruker shapes)
;different carrier positions in t1 and t2
;AV III instruments only!!
;requires dccorr off to be typed for shorter delays
;NS = N*8 to cancel artefacts
;Set:
;PULSES:
;p1  :  proton 90 degree pulse at pl12
;p4  :  proton 180d pulse at plw12
;p10 :  length of decoupling shapefile in t1
;p15 : selective excitation in z-Filter
;p5  :  PMLG cycle/2
;
;POWER LEVELS:
;plw1  : proton 90d pulse
;plw12 :  power level for initial excitation pulses
;plw13 : power level for PMLG in t2 (for simplicity: plw13 = plw12)
;
;DELAYS:
;d1  :  recycle delay
;d2  : dephasing delay for z-Filter
;d21 :  delay after RG_OFF (eg 0.3 us)
;d22 :  delay before RG_ON (deadtime, eg. 3.4 us)
;d25 :  theta1 in s = deg, orientation of eff. field for FSLG in t1
;
;CONSTANTS:
;cnst10 :  carrier offset for FSLG in t1 in Hz
;cnst11 :  carrier offset for wPMLG in t2 in Hz
;cnst20 :  rf field strength along thetaM for wPMLG in t2 (used to compute P5)
;
;COUNTER
;l11 : no. of oversampled datapoints in t2 (eg 6)
;$COMMENT=homonuclear decoupling with w-pmlg
;$CLASS=Solids
;$DIM=2D
;$TYPE=homonuclear decoupling
;$SUBTYPE=explicit acquisition
;$OWNER=Bruker
"acqt0=0"
"spw1=plw13" ;power level for PMLG in t2
dwellmode auto
#include <Avancesolids.incl>
#include <Delayssolids.incl>
#include <lgcalc.incl>
/*;set cnst20 to RF field at pl13, wpmlg pulse calculated as multiple of 100 ns */
"d9 = 0.1u*(l11)" ;set the sampling window in Avancedru.incl
;set initial value for d0 (t1 loop counter)
"d0 = 1.0u"
"l0 = 0"
;Pulses for FSLG decoupling in t1
"p3 = (d25/90)*p1" ;theta1 = orientation of eff. field during t1
"p2 = p1-p3" ;90d - theta1
;default delays
  "d60=0.1u"
  "d62=1.0u"
```

```
define delay dead
"dead = d22" ;delay before RG_ON (deadtime)
"p9 = dead+0.1u+d21+d9" ;total length of window in t2
define pulse smooth
define loopcounter polish
define pulse wpmlg
"smooth = 10*p5"  ;p5 computed from cnst20 (approx. rf power during PMLG)
"polish = 1u*smooth/10"
"wpmlg = 2u*polish" ;length of wPMLG shape in t2
define delay cycle
"cycle = p9+wpmlg" ;cycle time for wPMLG
define loopcounter count ;loop counter for data acquisition t2
define loopcounter nrows
"nrows = td1/2"
"count = aq/2*cycle-0.5" ;make sure td datapoints sampled in t2 (2 per loop)
define delay rest ;make sure sampling proceeds throughout the sequence
   "rest = aq-(2*count*cycle)" ;2*count*cycle is total actual acquisition time
   "blktr2 = 0.7u" ;opens transmitter gate 0.7 usec before pulse
;(transmitter noise not sampled)
;Compute spectral width that should be set
define loopcounter setswh
"setswh = 1/cycle" ;set swh2 to this value
define delay setdwell
"setdwell = 2/setswh"
; #----- START OF PULPROG
#include <fastCRAMPS.incl>
1 ze ;acquire into a cleared memory
   setdwell ;show calculated dwell and swh in F1
2 50m do:f1
  .1u fq=cnst10 :f1 ;set carrier for t1
  10u reset1:f1
; #--- Saturation
3 1u pl12:f1
  (p1 pl12 ph9):f1
  25m
  lo to 3 times 5
4 d1 pl12:f1        ;recycle delay
  "l0=d0/p10" ;increment l0 (t1)
; #--- Nutation-frequency-selective z-Filter
  (p1 pl12 ph1):f1 ;90d hard excitation
  (p15:sp15 ph21):f1 ;selective excitation (shaped pulse)
  d2 ;dephasing delay
; #--- Decoupling in t1 (with echo)
  (p1 pl12 ph0):f1    ;90d hard excitation 1H
  d60 pl20:f1
  d62
5  (p10:spf10 ph11):f1 ;FSLG (shaped pulse),echo time tau
  lo to 5 times l0 ;repeat l0 times
  .1u pl12:f1
      (p4 pl12 ph12):f1  ;180d refocusing pulse
  .1u pl20:f1  ;set power level back for FSLG
9 (p10:spf10 ph11):f1    ;FSLG again, echo time tau
  lo to 9 times l0 ;repeat l0 times
  d60 pl12:f1   ;same delays as before pi pulse
  d62
; #--- wPMLG detection in t2
  STARTADC ;prepare adc for sampling, set reference frequency, defined in Avancedru.incl
```

```
  RESETPHASE ;reset reference phase
  (p2 pl12 ph6):f1   ;90d-theta1 flip back
  (p1 pl12 ph8):f1   ;90d hard excitation
  .1u fq=cnst11 :f1  ;set carrier frequency for wPMLG in t2
  1u
  (p1 pl12 ph7):f1   ;90d hard excitation
  .1u DWL_CLK_ON
6 dead  pl13:f1
  d9 RG_ON ;receiver gain on for d9
  .1u RG_OFF             ;take l11  complex data points
  d21
  (wpmlg:sp1  ph10^):f1 ;wPMLG phase = x
  dead
  d9 RG_ON
  .1u RG_OFF
  d21
  (wpmlg:sp1  ph10^):f1 ;wPMLG phase = -x
  lo to 6 times count ;make sure td points are sampled
  rest
  1u  DWL_CLK_OFF
  rcyc=2             ;next scan
7 100m wr #0 if #0 zd   ;phase insensitive detection
  1u id0                ;increment t1
  lo to 2 times nrows
  exit ;finished
; #--- Phase cycles
ph9=0           ;saturation pulses
ph1=1 3         ;selective z filter (90d pulse)
ph21=0          ;selective excitation pulse
ph0=0   ;initial 90d
ph8=2
ph6=1           ;90d-theta1 flip back
ph7=0 0 1 1 2 2 3 3     ;90d before wPMLG
ph12=3         ;phase for pi pulse (echo in t1)
ph10=0 2   ;wPMLG in t2
ph11=0   ;FSLG in t1
ph31=0 2 1 3 2 0 3 1    ;receiver
```
